# The AP-1 transcription factors c-Jun and JunB are essential for CD8α conventional dendritic cell identity

**DOI:** 10.1038/s41418-021-00765-4

**Published:** 2021-03-23

**Authors:** Philipp Novoszel, Barbara Drobits, Martin Holcmann, Cristiano De Sa Fernandes, Roland Tschismarov, Sophia Derdak, Thomas Decker, Erwin F. Wagner, Maria Sibilia

**Affiliations:** 1 Institute of Cancer Research, Department of Medicine I, Comprehensive Cancer Center, Medical University of Vienna, Vienna, Austria; 2 Department of Microbiology, Immunobiology and Genetics, Max Perutz Labs, University of Vienna, Vienna, Austria; 3 Core Facilities, Medical University of Vienna, Vienna, Austria; 4 Department of Dermatology and Department of Laboratory Medicine, Medical University of Vienna, Vienna, Austria

**Keywords:** Development, Antigen-presenting cells, Antimicrobial responses, Bone marrow transplantation, Immunogenetics

## Abstract

Dendritic cell (DC) development is orchestrated by lineage-determining transcription factors (TFs). Although, members of the activator-protein-1 (AP-1) family, including Batf3, have been implicated in conventional (c)DC specification, the role of Jun proteins is poorly understood. Here, we identified c-Jun and JunB as essential for cDC1 fate specification and function. In mice, Jun proteins regulate extrinsic and intrinsic pathways, which control CD8α cDC1 diversification, whereas CD103 cDC1 development is unaffected. The loss of c-Jun and JunB in DC progenitors diminishes the CD8α cDC1 pool and thus confers resistance to Listeria monocytogenes infection. Their absence in CD8α cDC1 results in impaired TLR triggering and antigen cross-presentation. Both TFs are required for the maintenance of the CD8α cDC1 subset and suppression of cDC2 identity on a transcriptional and phenotypic level. Taken together, these results demonstrate the essential role of c-Jun and JunB in CD8α cDC1 diversification, function, and maintenance of their identity.

## Introduction

Dendritic cells (DCs) are innate immune cells essential for the initiation of antigen-specific immune responses and maintenance of tolerance [1]. DCs originate from multipotent, hematopoietic precursors in the bone marrow (BM) and can be categorized into two distinct branches, conventional (cDCs) and plasmacytoid DCs (pDCs), characterized by their unique transcriptional, functional, and cell surface phenotypes [2–4]. cDCs can be sub-grouped in cDC1, specialized in cross-presentation and cDC2, critical for the induction of T_h_2 and T_h_17 immunity [5]. cDC1 can be identified by the expression of X-C motif chemokine receptor 1 (XCR1) and comprise a lymphoid-resident CD8α^+^ and a non-lymphoid CD103^+^ population [6, 7]. Over the last decade studies have shown that the differentiation into cDC1 is controlled by a network of specific, lineage-determining transcription factors (TF) [8, 9]. TFs like the interferon regulatory factor 8 (IRF8), inhibitor of DNA binding 2 (ID2), and nuclear factor interleukin 3-regulated (NFIL3) are required for the diversification of the common DC progenitor (CDP) to cDC1 [10–12]. Furthermore, the basic leucine zipper transcription factor, ATF-like 3 (Batf3), a member of the dimeric activator protein-1 (AP-1) TF family, has been shown to maintain IRF8 expression in pre-cDCs, the direct clonogenic precursor to cDCs, to safeguard cDC1 differentiation [13, 14].

AP-1 TFs comprise a group of homo- or heterodimers of basic leucine zipper proteins belonging to the Jun, Fos, MAF or ATF family with essential functions in development, cancer and immunity [15–17]. c-Jun and JunB are AP-1 family members with prominent roles in T cell development [18] and T_h_17 identity [19], respectively. Moreover, JunB inactivation in stem cells results in a myeloproliferative disorder characterized by increased numbers of granulocyte/macrophage progenitors (GMP) [20, 21]. Functionally, c-Jun and JunB can be antagonistic but also compensatory, emphasizing that expression of some target genes is regulated equally by c-Jun and JunB. The inducible epidermal deletion of both c-Jun and JunB, for example, causes a psoriasis-like skin phenotype in adult mice, whereas single deletion results in no phenotype [22–25].

The interaction between Batf and Jun was originally considered to inhibit AP-1 transcriptional activity [26, 27]. However, a transcriptional complex between Jun/Batf and IRF4 or IRF8 enables binding to AP-1–IRF composite elements (AICEs), which is critical for the development of cDC1s [28] and the differentiation of T_h_17 cells [29, 30]. Although deletion of Batf3 in a genetically engineered mouse model (GEMM) demonstrated its role in CD8α/CD103 development, the importance of Jun proteins, is poorly defined. In this study, we show that combined, immune cell-specific deletion of c-Jun and JunB leads to the selective loss of lymphoid CD8α cDC1, whereas non-lymphoid CD103 cDC1 differentiate normally. Importantly, c-Jun and JunB are required for cDC1-dependent immune responses, including activation upon TLR triggering and antigen cross-presentation. Mechanistically, our data reveal for the first time that c-Jun and JunB maintain cDC1 identity and suppress expression of cDC2 TF and surface markers.

## Methods

### Animal studies

*c-Jun*^fl/fl^ and *JunB*^fl/fl^ mice were crossed with *CD11c*-Cre, *Mx*-Cre or Tamoxifen (Tx) inducible *K5*-Cre^ER^ mice, [31–34]. *Mx*-Cre mice received one or two (indicated in Fig. legends) intraperitoneal injections of poly I: C (200 µg; VWR) and *K5*-Cre^ER^ mice five consecutive injections of Tx (1 mg; Sigma Aldrich) for deletion. A 5% cream formulation of Imiquimod (Aldara, Meda Pharma) was applied topically on shaved and waxed (Veet, Heidelberg, Germany) back skin for up to 7 days [35]. *C57Bl/6 CD45.1, CD45.2 or CD45.1/CD45.2* were bred in house. Female and male mice of a mixed background (129 Sv × C57BL/6) and 8–16 weeks of age were used for studies. Mice were kept in the animal facility of the Medical University of Vienna in accordance with institutional policies and federal guidelines. All mice had access to food and water ad libitum.

### Flow cytometry

Single cell suspensions of lymphoid organs and BM were prepared as follows. BM was flushed, spleen and lymph nodes were digested for 30 min (37 °C) with Liberase™ (50 µg/mL; Roche) and DNAse I (100 µg/mL; Sigma Aldrich), filtered through a 70 µm filter and washed. Red blood cells were lysed with ACK buffer. Single cell suspensions were blocked with anti-CD16/32 antibody (BioLegend) before addition of fluorescently labeled antibodies. Antibodies (see Supplementary Table 1) were diluted according to the manufacturer´s recommendation and samples incubated on 4 °C for 30 min. Absolute cell number was assessed with a CASY cell counter (Beckman Coulter) or by addition of 123count eBeads (Thermo Fisher Scientific). Cells were acquired on a LSR Fortessa cell analyzer (BD Biosciences) and analyzed with FlowJo software (Treestar).

### Intracellular cytokine staining

For intracellular staining of IL-12 p40 (C15.6; BioLegend) cells were stimulated with poly I: C (1 µg/mL; VWR) in the presence of Brefeldin A (BioLegend) for 5 h. Surface markers were stained before fixation and permeabilization with the BD Cytofix/Cytoperm Kit (BD Biosciences). IRF4 (3E4; eBioscience) and IRF8 (V3GYWCH; eBioscience) were stained intracellular in spleen and lymph-node using the Foxp3 /Transcription Factor Staining Buffer Set (eBioscience).

### Cell culture

BM cells were isolated from femur/tibia and red blood cells were lysed. 3 × 10^6^ cells/mL were cultured in RPMI supplemented with 10% FCS, 1% Pen/Strep, sodium pyruvate, nonessential amino acids and 0.1% β-mercaptoethanol. Recombinant mFLT3L (80 ng/mL, Peprotech) was added to induce cDC and pDC differentiation. For CD103^+^ cDC in vitro differentiation BM cells were depleted of lineage-positive cells using the lineage cell depletion kit (Miltenyi Biotec). Enriched cells were cultured in complete medium supplemented with recombinant mFLT3L (80 ng/mL, Peprotech) and recombinant mGM-CSF (10 ng/mL, Peprotech) for 12 days. On day 6 fresh culture media supplemented with recombinant mFLT3L and mGM-CSF was added.

### Generation of bone marrow chimeric mice

BM chimeric mice were generated by intravenous *(i.v.)* injection of CD3ε/CD90.1.-MACS-depleted BM cells (4 × 10^6^) into lethally irradiated mice (8.25–9 Gy). For mixed BM chimeras, BM cells of two different donors (CD45.1/2 wild-type and CD45.2 *c-Jun*/*JunB* deficient) were mixed in a ratio 1:1 before injection. Experiments were performed after a reconstitution period of at least 8 weeks. Poly I: C was injected into chimeric mice twice, 5 days apart, and mice were analyzed 14 days after Cre induction.

### In vivo α-G-CSF antibody treatment

Mice were treated with 50 μg of α-GCSF IgG (clone 67604, Sigma Aldrich), or matched isotype control (rat IgG, BioXCell) by intraperitoneal injection 2 and 4 days after poly I: C induced deletion of c-Jun/JunB in the *Mx*-Cre mouse model.

### Listeria infection model

An overnight culture of *L. monocytogenes* strain LO28 was re-cultured in BHI medium to late log phase, pelleted and diluted in PBS. The concentration of bacteria was quantified by optical density measurements at 600 nm and confirmed by plating serial dilutions on BHI agar plates and colony counting. 1 × 10^6^ cfu were injected into the peritoneum of 8- to 15-week-old mice. Animals were euthanized after 24 and 72 h, spleens and livers were isolated, weighed and homogenized, and serial dilutions were plated on BHI agar plates. Colonies were counted after ~30 h culture at 37 °C.

### In vitro cross-presentation

*c-Jun/JunB* cDC^FL^ were obtained by negative selection of B220^+^ pDC^FL^ by MACS on day 7 of FLT3L-supplemented BM cultures. cDC^FL^ were then loaded with Ovalbumin (250 µg/mL) or SIINFEKL (20 µg/mL) for 6 h, washed and re-plated at 2 × 10^4^ with CFSE labelled (1 µM, 10 min) OT-I T cells in a 1:100 cDC^FL^ to OT-I T cell ratio. OT I- T cells were enriched from lymphoid organs of OT-I mice by negative depletion of B (CD19^+^, B220^+^), NK (NK 1.1^+^), DC (CD11c^+^) and myeloid (CD11b^+^) cells. After 3 days of co-culture, proliferation of OT-I T cells was analyzed by CFSE dilution in TCRβ^+^CD8α^+^ cells.

### Quantitative PCR

Total RNA was isolated with TRIzol reagent (Invitrogen) and cDNA synthesis was done with SuperScript IV Reverse Transcriptase (Thermo Fisher Scientific). Real-time PCR was performed with SYBR Green Master Mix (Applied Biosystems) on a CFX96 Touch System (BioRad). Differential expression is shown as fold change and was calculated with the ΔΔCt method. Expression was normalized to the house keeping gene *Tbp* (TATA-binding protein). Primers used for quantitative PCR are listed in Supplementary Table 2.

### RNA-sequencing

FLT3L-derived cDC1^FL^ (CD24^+^CD11c^+^B220^−^ CD115^−^ CD172a^−^) cells were sorted (FACSAria Fusion cell sorter; BD Biosciences) into TRIzol LS reagent (Invitrogen) and RNA was isolated with the miRNeasy Micro Cleanup Kit (Qiagen). Sequencing libraries were prepared using the NEBNext Poly (A) mRNA Magnetic Isolation Module and the NEBNext Ultra^™^ II Directional RNA Library Prep Kit for Illumina according to manufacturer’s protocols (New England Biolabs). Libraries were QC-checked on a Bioanalyzer 2100 (Agilent) using a High Sensitivity DNA Kit for correct insert size and quantified using Qubit dsDNA HS Assay (Invitrogen). Pooled libraries were sequenced on a NextSeq500 (Illumina) in 2 × 75 bp paired-end sequencing mode. Reads in fastq format were aligned to the murine reference genome version mm10 (https://www.ensembl.org) with Gencode vM19 annotations (https://www.gencodegenes.org/) using STAR aligner [36] version 2.6.1a in 2-pass mode. Reads per gene were counted by STAR, and differential gene expression was calculated using DESeq2 [37] version 1.20.0. Principal component analysis (PCA) and Euclidean distance plots were generated using R functions prcomp and dist, respectively. GO term enrichment analysis was performed with GOrilla [38] using as an input selections of differentially expressed genes (DEGs) and all mouse Ensemble gene ids as a background. Gene set enrichment analysis (GSEA) was performed using GSEA [39] version 3.0 using as an input regularized log-transformed count data from DESeq2 and selected gene sets from MSigDB [40] version 6.2 as well as custom gene sets.

### Venn diagram

To generate Venn diagrams we first made a list of probe sets derived from the published microarray data with the Transcriptome Analysis Software (Thermo Fisher Scientific). We then used the GSEA software (http://www.gsea-msigdb.org/gsea/index.jsp) to collapse the values of the probe sets to a single gene value by gene using the median probe mode. We considered only genes that were annotated on both, the microarray and RNA-Seq platform. We used the R function euler to generate a Venn diagram.

### Statistics

GraphPad Prism 8 was used for statistical analysis. Significance was analyzed on pooled data from independent experiments. Animals were randomly assigned to experimental groups. Data were analyzed by unpaired two-tailed Students *t* test or one-way ANOVA with Tukey post-test, or multiple *t*-tests with the Holm–Sidak method for grouped data and are shown as mean ± SEM. *t*-test with Welch´s correction was performed, if variance was significantly different between two sets of data. Outliers were identified by Grubb´s or ROUT method. A *P* value below 0.05 was considered statistically significant (*P < 0.05, **P < 0.01, ***P < 0.001, ****P < 0.0001).

## Results

### Combined deletion of c-Jun and JunB results in loss of resident CD8α cDC1, while migratory CD103 cDC1 remain unaffected

To study the role of Jun/AP-1 proteins during DC diversification, we first conducted an unbiased gene expression analysis of c-Jun and JunB across the immune cell compartment using IMMGEN datasets. We found prominent expression of c-Jun and JunB in cDCs, but not in pDCs (Supplementary Fig. 1a).

**Fig. 1:**
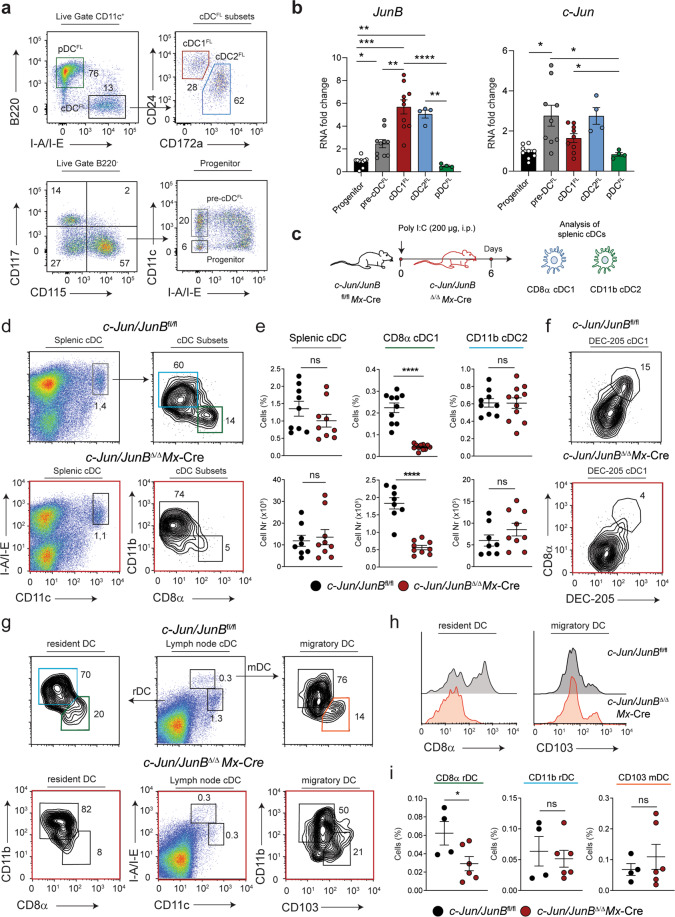
Combined deletion of c-Jun and JunB results in loss of resident CD8α cDC1, while migratory CD103 cDC1 remain unaffected. **a** Representative flow cytometry plots depict the sorting strategy for pDCs^FL^ (CD11c^+^ B220^+^), the cDC ^FL^ subsets (CD11c^+^ I-A/I-E^+^): cDC1^FL^ (CD24^+^ cDC^FL^), cDC2^FL^ (CD172a^+^ cDC^FL^), pre-cDCs^FL^ (B220^-^ CD117^low-int^ CD115^+^ CD11c^+^ I-A/I-E^-^), and progenitors (B220^-^ CD117^low-int^ CD115^+^ CD11c^-^ I-A/I-E^-^) generated from wild-type bone marrow supplemented with FLT3L for 8d. **b** RT-qPCR analysis of *c-Jun* and *JunB* mRNA expression in progenitors, pre-cDC^FL^, cDC1^FL^, cDC2^FL^, pDC^FL^. Subsets were obtained according to (**a**). RNA fold change is shown relative to the progenitor population. **c** Experimental design. Deletion of c-Jun and JunB in the *Mx*-Cre mouse model was induced by injection of poly I: C (i.p., 200 µg). Mice were analyzed 6 days after the first injection. **d** Representative flow cytometry plots show single, live splenic cDCs (CD11c^+^I-A/I-E^+^) and CD8α cDC1 (CD8α^+^ CD11b^-^ cDC) and CD11b cDC2 (CD8α^-^ CD11b^+^ cDC) subsets from indicated mice. **e** Frequency and number of splenic CD8α cDC1 and CD11b cDC2 as defined in (**d**) are shown. **f** Representative flow cytometry plots depict splenic cDC1 defined as DEC-205^+^CD11c^+^ cells in control *c-Jun/JunB* ^fl/fl^ and in *c-Jun/JunB* ^Δ/Δ^*Mx*-Cre mice. **g** Skin-draining lymph node (sd-LN) cDCs consisting of migratory DCs (mDCs; CD11c^+^I-A/I-E^high^) and resident DCs (rDCs; CD11c^+^I-A/I-E^int^) were analyzed by flow cytometry for the CD8α rDC and CD103 mDC subset in *c-Jun/JunB* ^fl/fl^ and *c-Jun/JunB* ^Δ/Δ^*Mx*-Cre mice. **h** Flow cytometric histograms show CD8α or CD103 expression on sd-LN cDCs (CD11c^+^I-A/I-E^int-high^) in the indicated mice. **i** rDCs and mDCs from (**g**) are shown as percentage of live, single cells. Data are representative of 2–5 independent experiments. Dots indicate number of individual mice per experimental group. Error bars represent mean ± SEM. Statistical significance was determined by Brown–Forsythe and Welch ANOVA test (**b**) and unpaired two-tailed Student’s *t* test (**e**, **i**). ****P < 0.0001, ***P < 0.001, **P < 0.01, *P < 0.05 and ns > 0.05.

To confirm these results, FLT3L-derived cDCs (cDCs^FL^) were generated from BM to analyze the expression of c-Jun and JunB during DC development (Fig. 1a). RNA expression from sorted cell subpopulations revealed an increase in JunB expression from progenitors to cDCs^FL^, whereas levels remained low in pDCs^FL^. In contrast, c-Jun mRNA levels were highest in pre-cDCs^FL^ and cDC2^FL^, intermediate in cDC1^FL^ and low in pDC^FL^ (Fig. 1b). Analysis of c-Jun and JunB expression in cDCs, pre-cDCs and CDPs in vivo, using previously published microarray data [14], confirmed these results (Supplementary Fig. 1b).

To determine, whether these Jun/AP-1 factors are functionally involved in DC development, *c-Jun*^fl/fl^ and *JunB*^fl/fl^ mice were crossed with *Mx*-Cre mice allowing induction of Cre-recombinase in a diverse set of cells, including the hematopoietic stem cell compartment, by injection of poly I: C [31, 32, 34]. No differences in splenic cDC1 and cDC2 subsets, identified by CD8α and CD11b marker expression among cDCs, were observed in *c-Jun*^Δ/Δ^*Mx*-Cre and *JunB*^Δ/Δ^*Mx*-Cre mice (Supplementary Fig. 1c-f). Thus, although c-Jun and JunB are induced during cDC development, these Jun/AP-1 proteins individually are dispensable for cDC diversification.

Closely related AP-1 proteins can functionally substitute for the absence of a family member during development and proliferation [24]. We speculated that c-Jun and JunB might compensate for each other, so that only combined deletion of both AP-1 factors would affect DC diversification.

Loss of c-Jun/JunB in *c-Jun/JunB*^Δ/Δ^*Mx-*Cre mice (Fig. 1c) induced a dramatic reduction in the frequency and total cell number of splenic CD8α cDC1, whereas numbers of CD11b cDC2 were unchanged (Fig. 1d, e). DEC-205 expression correlates with CD8α on cDC1s [41]. The DEC-205^+^CD11c^+^ population in *c-Jun/JunB*^Δ/Δ^*Mx*-Cre mice was reduced, confirming that the observed phenotype in cDC1 cells was not caused by down-regulation of CD8α on cDCs (Fig. 1f). Moreover, changes in the splenic immune cell compartment, in particular myeloid cells (CD11b^+^Ly-6C/G^+^), could be observed. The frequency of splenic B and T cells was altered, the absolute number, however, was not affected (Supplementary Fig. 2a, b). Interestingly, these observed changes in the immune cell composition preceded a more severe phenotype that developed within 2–3 weeks of Cre recombinase induction and included an inflamed skin (Supplementary Fig. 2c), weight loss and increased mortality (data not shown).

We then examined cDC populations in skin-draining lymph-nodes (sd-LN) of *c-Jun/JunB*^Δ/Δ^*Mx*-Cre. Reduced populations of both lymphoid-resident (CD8α) and migratory (CD103) cDC1 have been reported in IRF8*-* and Batf3-deficient mice [42]. However, in *c-Jun/JunB*^Δ/Δ^*Mx*-Cre mice only the resident CD8α cDC1 population was significantly reduced (Fig. 1g–i).

In c-*Jun/JunB*^Δ/Δ^*Mx*-Cre mice deletion of c-Jun/JunB occurs already in hematopoietic stem cells and might affect the commitment of multipotent progenitors to a single fate. We detected a significant expansion of granulocyte-macrophage progenitors (GMPs) in the BM of *c-Jun/JunB*^Δ/Δ^*Mx-*Cre mice, whereas progenitors, precursors of cDCs and lymphocytes were unchanged (Supplementary Fig. 2d-f).

To better characterize cDC development from BM progenitors, we cultured c-Jun/JunB deficient BM cells supplemented with FLT3L. We observed prominent defects in cDC1^FL^ differentiation and maturation in c-Jun/JunB-deficient BM cells (Supplementary Fig. 2 g–j).

Taken together, these data imply that c-Jun and JunB have an overlapping function and are dispensable for early DC development, but crucial for the terminal differentiation to CD8α cDC1.

### Intrinsic and extrinsic factors determine the fate of CD8α cDC1 in* c-Jun/JunB*^Δ/Δ^*Mx*-Cre mice

**Fig. 2:**
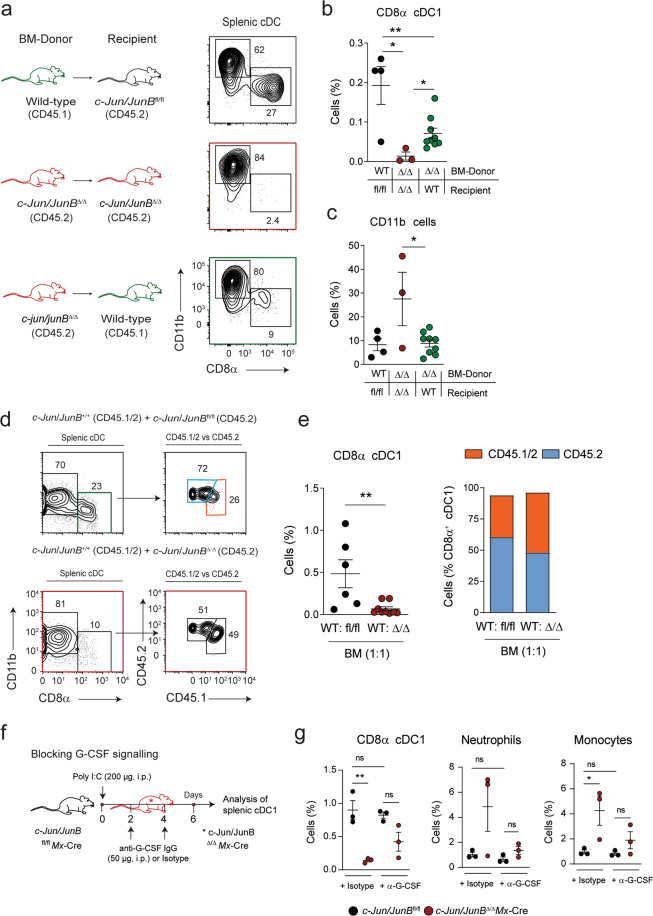
Intrinsic and extrinsic factors determine the fate of CD8α cDC1 in *c-Jun*/*JunB*^Δ/Δ^*Mx*-Cre mice. **a** Experimental design and representative flow cytometry plots of splenic cDCs (CD11c^+^I-A/I-E^+^) for the different BM chimeras are shown. Donor BM (2–4 × 10^6^) of the indicated genotypes was injected *i.v*. into lethally irradiated wild-type, *c-Jun*/*JunB*^fl/fl^ and *c-Jun*/*JunB*^fl/fl^*Mx*-Cre recipient mice. Deletion was induced by injection of poly I: C (2 times, 5 days apart) and mice were analyzed 14 days after. **b–c** Splenic CD8α cDC1 (CD11c^+^CD8α^+^) (**b**) and CD11b cells  (CD11b^+^) (**c**) were analyzed by flow cytometry in BM chimeras described in (**a**). **d** Representative flow cytometry plots show splenic cDCs generated from mixed BM chimeras. Wild-type, CD45.1 and CD45.2 expressing BM, was mixed in a 1:1 ratio with CD45.2 *c-Jun*/*JunB*^fl/fl^ or *c-Jun*/*JunB*^fl/fl^*Mx*-Cre BM. Deletion was induced by injection of poly I: C (2 times, 5 days apart) and mice were analyzed 14 days after. **e** Splenic cDCs from (**d**) were analyzed for the percentage of CD8α cDC1 (CD11c^+^CD8α^+^) (left)  and their  expression of CD45.1/2 or CD45.2 (right). **f** Scheme depicting the treatment to neutralize G-CSF signaling in *c-Jun*/*JunB*^Δ/Δ^*Mx*-Cre mice. Cre recombinase was induced by injection of poly I: C (200 µg, i.p.). Anti-G-CSF IgG antibodies (50 µg, i.p.) were injected 2 and 4 days after deletion of c-Jun/JunB. Isotype was injected in controls. Analysis of splenic cDC1 and myeloid cells was done on day 6 post poly I: C injection. **g** Splenic CD8α cDC1 (CD11c^+^MHCII^+^XCR1^+^CD8α^+^), monocytes (Ly6-C^+^CD11b^+^) and neutrophils (Ly6G^+^ CD11b^+^) were analyzed in mice of the indicated genotype treated as described in (**f**). Data are representative of 1–3 independent experiments. Flow cytometry plots shown are pre-gated on single, live cells. Dots indicate number of individual mice per experimental group. Error bars represent mean ± SEM. Statistical significance was determined by unpaired two-tailed Student’s *t* test (**b**, **c** and **e**) and one-way ANOVA with Tukey post-test (**g**). **P < 0.01, *P < 0.05 and ns > 0.05.

To determine, if the diminished CD8α cDC1 population in *c-Jun/JunB*^Δ/Δ^*Mx-*Cre mice was due to cell-intrinsic effects of c-Jun/JunB in DCs or a consequence of extrinsic c-Jun/JunB dependent signals BM chimeras were generated. Loss of c-Jun/JunB only in hematopoietic cells partially rescued the splenic cDC1 phenotype after Cre recombinase activation, when compared to control mice (Fig. 2a, b). Thus, extrinsic and intrinsic factors are critical for CD8α cDC1 specification. In contrast, the increase in myeloid cells was rescued, if c-Jun/JunB was deleted only in BM cells (Fig. 2c).

To specify, if extrinsic signals from hematopoietic cells determine CD8α cDC1 fate competitive, mixed BM chimeras were generated by giving wild-type (CD45.1/2) and *c-Jun*/*JunB*^fl/fl^ or *c-Jun*/*JunB*^fl/fl^*Mx*-Cre (CD45.2) BM cells in a ratio of 1:1 into *c-Jun*/*JunB*^fl/fl^ mice. After recombination by poly I: C, the overall frequency of CD8α cDC1 was significantly reduced in mice harboring wild-type and *c-Jun/JunB*^Δ/Δ^*Mx-*Cre BM (Fig. 2d, e, left panel). The few remaining CD8α cDC1 were equally derived from both, wild-type and c-Jun/JunB-deficient BM (Fig. 2e, right panel).

Among other organs *Mx*-Cre activation also results in genomic recombination in skin cells. To investigate, whether extrinsic factors influence CD8α cDC1 differentiation we used a mouse model with deletion of c-Jun/JunB restricted to keratinocytes (*c-Jun/JunB*^Δ/Δ^*K5-*Cre^ER^) that results in a psoriasis-like skin inflammation [25, 43]. Splenic CD8α cDC1 were diminished in *c-Jun/JunB*^Δ/Δ^*K5-*Cre^ER^ mice, although not to the extent as in *c-Jun*/*JunB*^Δ/Δ^*Mx*-Cre mice (Supplementary Fig. 3a-c). In contrast, we did not observe a reduction in the Imiquimod induced psoriasis-like skin inflammation model (Supplementary Fig. 3d, e).

G-CSF, a JunB target gene [44], has been shown to influence cDC1 development [45]. To test the role of G-CSF signaling in our observed phenotype, we treated *c-Jun/JunB*^Δ/Δ^*Mx-*Cre with anti-G-CSF neutralizing antibodies in vivo (Fig. 2f). We found that suppression of G-CSF signaling partially restored splenic cDC1 in *c-Jun/JunB*^Δ/Δ^*Mx-*Cre mice and normalized the frequency of monocytes and neutrophils (Fig. 2g).

In summary, these data show that extrinsic and intrinsic mechanisms controlled by c-Jun/JunB have synergistic effects and together cause a severe reduction of cDC1 in *c-Jun*/*JunB* ^Δ/Δ^*Mx*-Cre mice.

### c-Jun and JunB coordinate cDC1 subset differentiation under homeostatic conditions

**Fig. 3:**
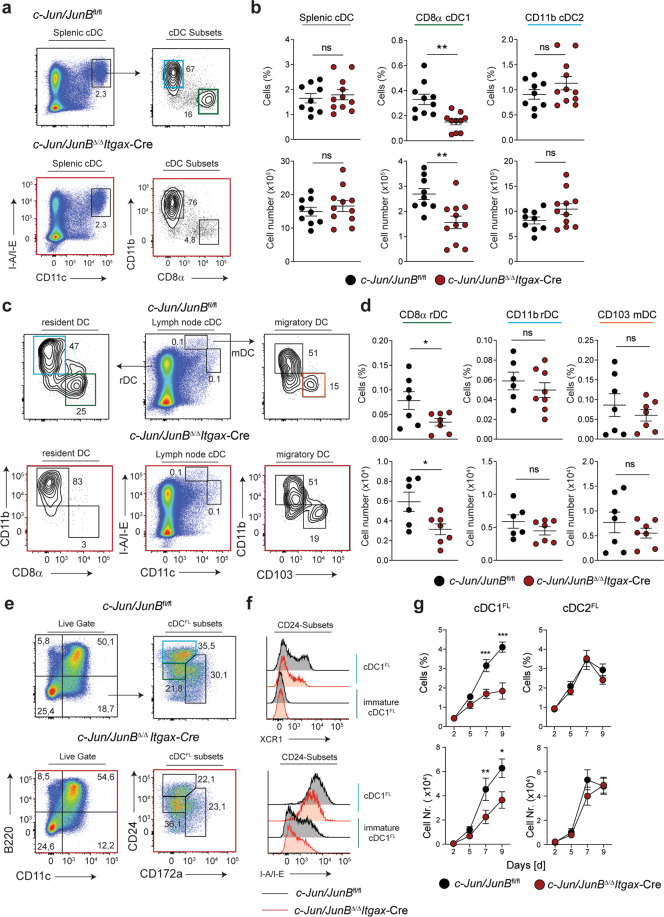
c-Jun and JunB coordinate cDC1 subset differentiation under homeostatic conditions. **a** Spleen from *c-Jun*/*JunB*^fl/fl^ and *c-Jun*/*JunB*^Δ/Δ^*Itgax*-Cre mice was analyzed by flow cytometry for cDCs (CD11c^+^I-A/I-E^+^), CD8α cDC1 (CD8α^+^ CD11b^−^ cDC), and CD11b cDC2 (CD8α^−^CD11b^+^ cDC). **b** Frequency and total numbers of splenic cDC subsets described in (**a**) are shown. **c** Representative flow cytometry plots of resident (CD11c^+^I-A/I-E^int^) and migratory DCs (CD11c^+^ I-A/I-E^high^) in skin-draining lymph nodes (sd-LN) of indicated mice are shown. **d** Frequency and total number of CD8α rDC, CD11b rDC and CD103 mDC sd-LN cDC subsets described in (**c**) are shown. **e** Representative flow cytometry plots of cDC1^FL^ (B220^−^CD11c^+^CD24^+^), immature cDC1^FL^ (B220^−^CD11c^+^CD24^int^) and cDC2^FL^ (B220^−^CD11c^+^CD172a^+^) in FLT3L-supplemented BM cultures of *c-Jun/JunB*^fl/fl^ and *c-Jun/JunB*^Δ/Δ^*Itgax*-Cre mice on day 7. **f** Histograms show XCR1 and I-A/I-E expression on cDC1^FL^ and immature cDC1^FL^ as defined in (**e**). **g** Frequency and total number of cDC1^FL^ and cDC2^FL^ in FLT3L-supplemented BM cultures over time. (*n* = 6–12 per subgroup). Data are representative of 2–4 independent experiments. Flow cytometry plots shown are pre-gated on single, live cells. Dots indicate number of individual mice per experimental group. Error bars represent mean ± SEM. Statistical significance was determined by unpaired two-tailed Student’s *t* test (**b**, **d** and **g**). ***P < 0.001, **P < 0.01, *P < 0.05 and ns > 0.05.

To further investigate the cell-intrinsic function of c-Jun/JunB within committed cDCs, we conditionally inactivated c-Jun/JunB in all CD11c^+^ cells by crossing *c-Jun*/*JunB*^fl/fl^ into *Itgax* (CD11c)-Cre mice. Deletion of c-Jun/JunB in CD11c^+^ cells did not lead to any apparent inflammatory phenotype. Adult *c-Jun/JunB*^Δ/Δ^*Itgax*-Cre mice (<8 weeks) showed normal frequencies of B cells, CD4 and CD8 T cells, myeloid cells, NK-cells and pDCs in the spleen (Supplementary Fig. 4a). However, loss of c-Jun/JunB at the pre-cDC stage induced a significant reduction of CD8α cDC1 (Fig. 3a, b) implying a phenotype similar to *c-Jun/JunB*^Δ/Δ^*Mx-*Cre mice. The overall reduction in CD8α cDC1 was less severe than in *c-Jun/JunB*^*Δ/Δ*^*Mx-*Cre mice and more comparable to WT + Δ/Δ BM chimeras (Fig. 2b). Other markers (XCR1, CD24 and DEC-205) [6] that identify the splenic cDC1 subset were diminished in the spleen, while the frequency of CD11b cDC2  was not altered (Fig. 3a, b, Supplementary Fig. 4b). Consistent to our data in the *Mx*-Cre mouse model, skin-draining LNs of *c-Jun/JunB*^Δ/Δ^*Itgax-*Cre mice showed a reduced number of CD8α rDC, whereas the CD103 mDC subset was not affected (Fig. 3c, d). Moreover, *c-Jun/JunB*^Δ/Δ^*Itgax*-Cre mice showed normal development of cDC- and myeloid progenitors in the BM, including pre-cDC1 and pre-cDC2 subpopulations (Supplementary Fig. 4c-e). These results further support our hypothesis that c-Jun and JunB together promote the differentiation of pre-cDCs to cDC1s under homeostatic conditions.

In vitro, significantly less cDC1^FL^ developed from *c-Jun/JunB*^Δ/Δ^*Itgax-*Cre BM. However, a prominent CD24^int^ population was seen in *c-Jun/JunB*^Δ/Δ^*Itgax-*Cre FLT3L BM cultures that lacked expression of typical DC markers, like XCR1 or MHC-II, resembling an immature cDC1^FL^ population (Fig. 3e-f). In contrast, normal differentiation of cDC2^FL^, pDC^FL^ and CDP^FL^ populations was observed, whereas pre-cDCs^FL^ were reduced after one week of culture (Fig. 3e, g and Supplementary Fig. 4f, g). Thus, c-Jun/JunB are required cell-intrinsically to orchestrate the final step of cDC1 development, the transition of pre-cDCs to cDC1, both in vitro and in vivo.

### GM-CSF signaling secures development of c-Jun/JunB-deficient CD103 cDC1

**Fig. 4:**
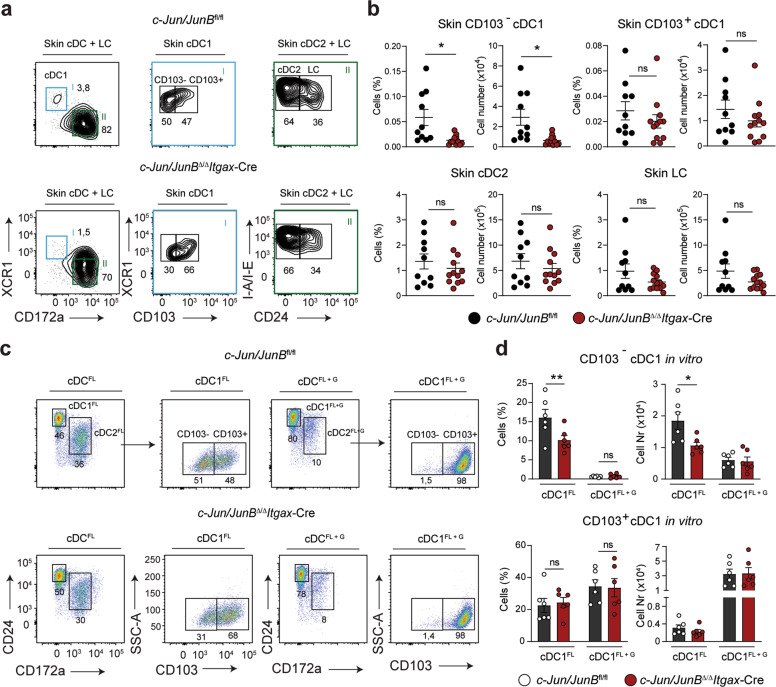
GM-CSF signaling secures development of c-Jun/JunB*-*deficient CD103 cDC1. **a** Skin from *c-Jun*/*JunB*^fl/fl^ and *c-Jun*/*JunB*^Δ/Δ^*Itgax*-Cre mice was analyzed by flow cytometry for cDCs (CD45^+^CD64^−^CD11c^+^I-A/I-E^+^), CD103^−^ cDC1 (XCR1^+^ CD103^−^ CD11b^−^ cDCs), CD103^+^ cDC1 (XCR1^+^ CD103^+^ CD11b^−^ cDCs), cDC2 (XCR1^−^ CD11b^+^ CD24^−^ cDCs) and LCs (XCR1^−^ CD11b^+^ CD24^+^ cDCs). Representative plots are shown and graphs are pre-gated on single, live cell populations. **b** Frequency and total numbers of cutaneous CD103^−^ and CD103^+^ cDC1, cDC2 and LCs as defined in (**a**) are shown. **c** Representative flow cytometry plots of cDCs (B220^−^ Gr-1^−^ CD11c^+^ MHC-II^+^) generated from FLT3L (cDC^FL^) or FLT3L + GM-CSF (cDC^FL + G^) supplemented BM cell cultures of the indicated genotypes. Gating for CD103^−^ and CD103^+^ cDC1 (CD24^+^CD172a^−^ cDCs) and cDC2 (CD24^−^CD172a^+^ cDCs) in vitro is shown. **d** Frequency and total numbers of in vitro generated CD103^−^ or CD103^+^ cDC1^FL^ or cDC1^FL+ G^ described in (**c**) are shown. Data are representative of 2–3 independent experiments. Dots indicate number of individual mice per experimental group. Error bars represent mean ± SEM. Statistical significance was determined by unpaired two-tailed Student’s *t* test (**b**) and multiple *t*-test with the Holm–Šídák method (**d**). **P < 0.01, *P < 0.05 and ns > 0.05.

Next, we analyzed cDCs in the skin, which contains two distinct cDC1 subsets with a CD103^−^ or CD103^+^ phenotype [46]. We found that cutaneous CD103^−^ cDC1 were reduced in *c-Jun/JunB*^Δ/Δ^*Itgax*-Cre mice, whereas CD103^+^ cDC1, cDC2 and Langerhans cells (LCs) were unchanged (Fig. 4a, b). Next, we analyzed lung and colon, two non-lymphoid tissues that contain a prominent CD103 cDC1 population. We observed no difference in CD103 cDC1 cells in *c-Jun/JunB*^Δ/Δ^*Itgax*-Cre compared to control mice (Supplementary Fig. 5a, b). GM-CSF controls non-lymphoid CD103 cDC1 development [47] and is used for the efficient generation of CD103 cDC1 from BM in vitro [48]. We therefore next analyzed CD103^−^ and CD103^+^ cDC1 generated from BM cultures supplemented with FLT3L alone or in combination with GM-CSF. cDCs deficient in c-Jun/JunB showed a decreased frequency in CD103^−^ cDC1^FL^, whereas CD103^+^ cDC1^FL^ and CD103^+^ cDC1^FL+G^ were comparable to the control (Fig. 4c, d). These data show that the GM-CSF-dependent CD103 cDC1 subset develops normally in *c-Jun/JunB*^Δ/Δ^*Itgax*-Cre mice.

### Global transcriptional changes in c-Jun/JunB deficient cDC1

**Fig. 5:**
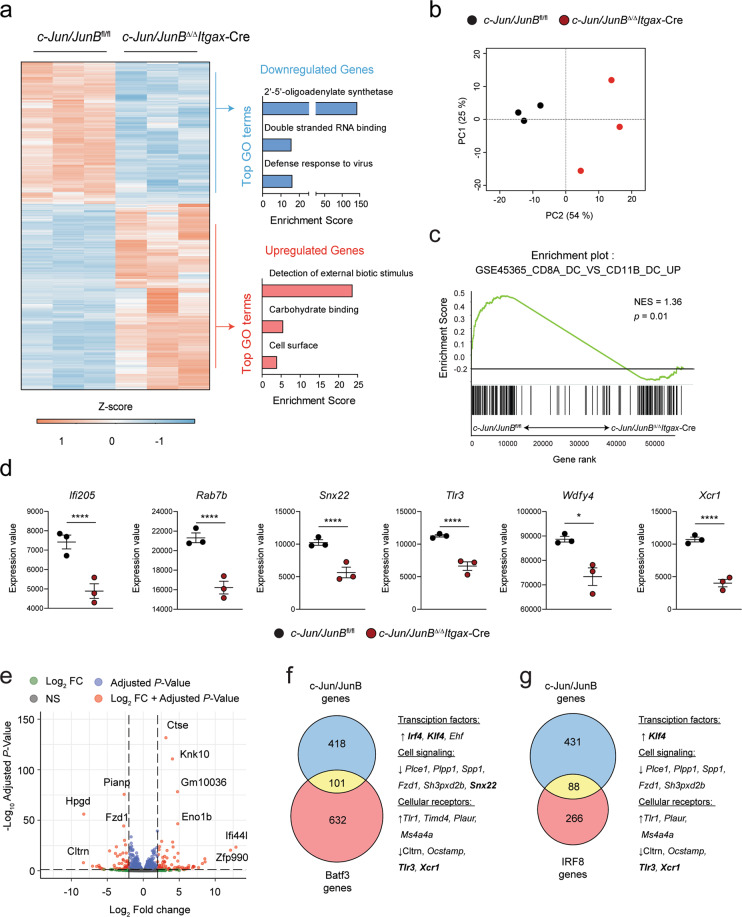
Global transcriptional changes in c-Jun/JunB deficient cDC1. **a** RNA Seq was performed on FACS sorted cDC1^FL^ (CD24^+^CD11c^+^B220^−^ CD115^−^ CD172a^−^) from indicated genotypes on day 8 of FTL3L supplemented BM cultures. Heat map shows differentially expressed genes (DEG) (*P* < 0.05, log_2_ FC ≥ 1.0). Gene ontology (GO) analysis was performed on differentially expressed genes and selected, enriched GO terms are shown (*P* < 0.05). **b** Principal component analysis (PCA) of control and c-Jun/JunB deficient cDC1^FL^ from RNA-Seq data generated as described in (**a**). **c** Gene set enrichment analysis (GSEA) for genes up-regulated in CD8α^+^ DC to CD11b^+^ DCs (MSigDb v 6.2; Gene set: GSE45365_CD8A_DC_VS_CD11B_DC_UP) comparing control to c-Jun/JunB deficient cDC1^FL^. **d** Graphs show RNA expression of cDC1-specific transcripts in *c-Jun/JunB*^fl/fl^ and *c-Jun/JunB*^Δ/Δ^*Itgax*-Cre cDC1^FL^ RNA-Seq samples. **e** Volcano plot depicting DEG in c-Jun/JunB deficient cDC1^FL^ cells. **f–g** Venn diagrams show the overlap of deregulated genes in *c-Jun/JunB* deficient cDC1^FL^ (abs (log_2_ FC ≥ 0.5); c-Jun/JunB genes) to *Batf3*^−/−^ splenic cDC1 (abs (log_2_ FC ≥ 1); Batf3 genes) [GSE40647] (**f**) or to *Irf4*^−/−^*Irf8*^−/−^cDC1^FL^ expressing low versus high IRF8 (abs(log_2_ FC ≥ 1); IRF8 genes) [GSE140451] (**g**). Selected genes of interest which are deregulated in both experiments are listed on the side. Data are from an RNA-Seq experiment with *n* = 3 per experimental group. Error bars represent mean ± SEM. Statistical significance was determined by unpaired two-tailed Student’s *t* test (**d**). ****P < 0.0001, *P < 0.05.

Next, we performed RNA sequencing to characterize the transcriptional profile in c-Jun/JunB deficient cDC1 in an unbiased manner. We compared in vitro generated cDC1s^FL^ from *c-Jun/JunB*^*Δ/Δ*^*Itgax*-Cre to *c-Jun/JunB*^fl/fl^ mice and found 432 genes to be differentially expressed (Supplementary Table 3). Gene ontology (GO) term analysis of the DEGs revealed down-regulation of genes linked to antiviral responses, like double-stranded RNA binding and 2′-5′-oligoadenylatesynthetase in c-Jun/JunB-deficient cDC1s^FL^, whereas genes linked to cell surface expression, enabling carbohydrate binding or detection of external stimuli, were up-regulated (Fig. 5a). Principal component analysis (PCA) further confirmed a clear segregation of c-Jun/JunB-deficient cDC1s^FL^ from control cDCs (Fig. 5b) and GSEA showed significant enrichment of cDC1 specific gene sets in the control only (Fig. 5c). Moreover, previously identified cDC1-specific genes (e.g., *Ifi205*, *Rab7b, Snx22, Tlr3, Wdfy4* and *Xcr1*) [49, 50] were all reduced in c-Jun/JunB-deficient cDC1s^FL^ (Fig. 5d). Among the top-ranked DEGs we found genes that are involved in functional processes, like the prostaglandin inactivating enzyme *Hpgd*, or a protease, *Ctse*, involved in antigen processing, a gene related to glycolysis (*Eno1b*), to interferon-signaling (*Ifi44l*) and an uncharacterized zinc finger transcription factor (*Zfp990*) (Fig. 5e).

To define genes that are common or unique to c-Jun/JunB, we compared our RNA-Seq data to published microarray data of *Batf3*^*−/−*^ cDC1 (Batf3 genes) and *Irf4*^−/−^*Irf8*^*−/−*^ cDC1^FL^ with restored IRF8 expression (IRF8 genes) [28, 49]. Among others, transcripts for the cDC2-associated TFs *Irf4, Klf4* and *Ehf* [51] were increased in both c-Jun/JunB and Batf3 deficient cDC1, whereas transcripts associated with cDC1 (*Snx22*, *Tlr3*, *Xcr1*) or cellular signaling (*Plce1*, *Plpp1, Spp1, Fzd1*) were reduced (Fig. 5f and Supplementary Table 4). Similar overlaps were observed comparing c-Jun/JunB to IRF8-dependent transcripts (Fig. 5g and Supplementary Table 5). These results show that c-Jun/JunB play an essential role to promote the gene expression in cDC1.

### cDC1 lacking c-Jun/JunB are functionally impaired in immune defense mechanisms

Our RNA-Seq analysis highlighted a role for c-Jun/JunB in the expression of genes (*Tlr3*, *Wdfy4*) and pathways (ds-RNA binding) associated with cDC1 function. Hence, we next investigated, whether these transcriptional changes impaired typical immune-functions of cDC1 in vivo and in vitro.

**Fig. 6:**
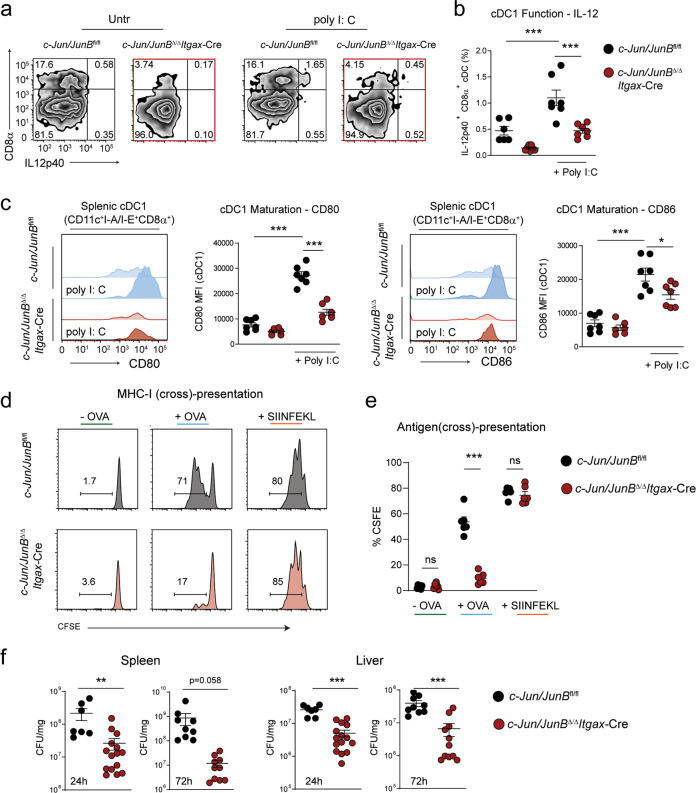
cDC1 lacking c-Jun/JunB are functionally impaired in immune defense mechanisms. **a** Splenic cDCs from *c-Jun/JunB*^fl/fl^ and *c-Jun/JunB*^Δ/Δ^*Itgax*-Cre mice were enriched by positive selection for CD11c and stimulated with the TLR3 ligand poly I: C (1 µg/mL) for 5 h in the presence of Brefeldin A ex vivo. Intracellular flow cytometry was performed to analyze IL-12 p40 production by CD8α cDC1. Representative plots shown are pre-gated on single, live, and cDCs (CD11c^+^I-A/I-E^+^). **b** Graph shows percentage of IL-12p40 producing cDC1 as defined in (**a**) to live, single cells. **c** Surface expression of CD80 and CD86 was analyzed on splenic cDCs by flow cytometry. cDCs were isolated from *c-Jun/JunB*^fl/fl^ and *c-Jun/JunB*^Δ/Δ^*Itgax*-Cre spleen with CD11c MicroBeads and stimulated with the TLR3 ligand poly I: C (1 µg/mL) for 16 h ex vivo. Representative histograms (left) and mean fluorescence intensity (MFI) (right) of CD80 and CD86 expression on cDC1 (CD11c^+^ I-A/I-E^+^ CD8α^+^) are shown. **d** cDC^FL^ were negatively selected from pDC^FL^ by depletion of B220^+^ cells by MACS. cDC^FL^ were then pulsed with Ovalbumin or SIINFEKL peptide and cultured with CFSE-labeled OT-I T cells at a ratio of 1:100 (DC: T cell ratio). After 3 days of co-culture, OT-I T cell proliferation (dilution of CFSE) was analyzed on live, single TCR-β^+^CD8α^+^ T cells by flow cytometry. Representative histograms are shown. **e** Graph shows proliferation of OT-I T cells, analyzed as described in (**d**) given as the percentage of CFSE dilution. **f** Listeria CFUs in spleen and liver of *c-Jun/JunB* ^fl/fl^ and *c-Jun/JunB* ^Δ/Δ^*Itgax*-Cre mice infected *i.v*. with *L. monocytogenes* (1 × 10^6^ per mouse) at the indicated time points. Data are representative of two independent experiments. Dots indicate number of individual mice per experimental group. Error bars represent mean ± SEM. Statistical significance was determined by unpaired two-tailed Student’s *t* test (**e**, **f**), or one-way ANOVA with Tukey post-test (**b**, **c**). ***P < 0.001, **P < 0.01, *P < 0.05, ns > 0.05.

Importantly, we found that splenic CD8α cDC1 and in vitro generated cDC1^FL^ lacking c-Jun/JunB expressed significantly less IL-12p40 and failed to up-regulate the co-stimulatory molecules CD80 and CD86 after stimulation with the TLR3 ligand poly I: C (Fig. 6a-c, Supplementary Fig. 5c-d). Moreover, antigen cross-presentation was impaired in the absence of c-Jun/JunB in cDCs^FL^ and a significantly reduced antigen-specific (Ovalbumin) CD8α OT-I T cell proliferation was induced, when compared to control (Fig. 6d, e). CD8α cDC1 have been shown to be a cellular entry point for infection with *Listeria monocytogenes* [52]. We observed a significantly reduced pathogen burden in the spleen and liver of *c-Jun/JunB*^Δ/Δ^*Itgax*-Cre mice infected with *L. monocytogenes* (Fig. 6f). Hence, these data suggest that c-Jun/JunB are important regulators for the functional properties of cDC1 in immune responses.

### c- Jun and JunB maintain cDC1 identity

**Fig. 7:**
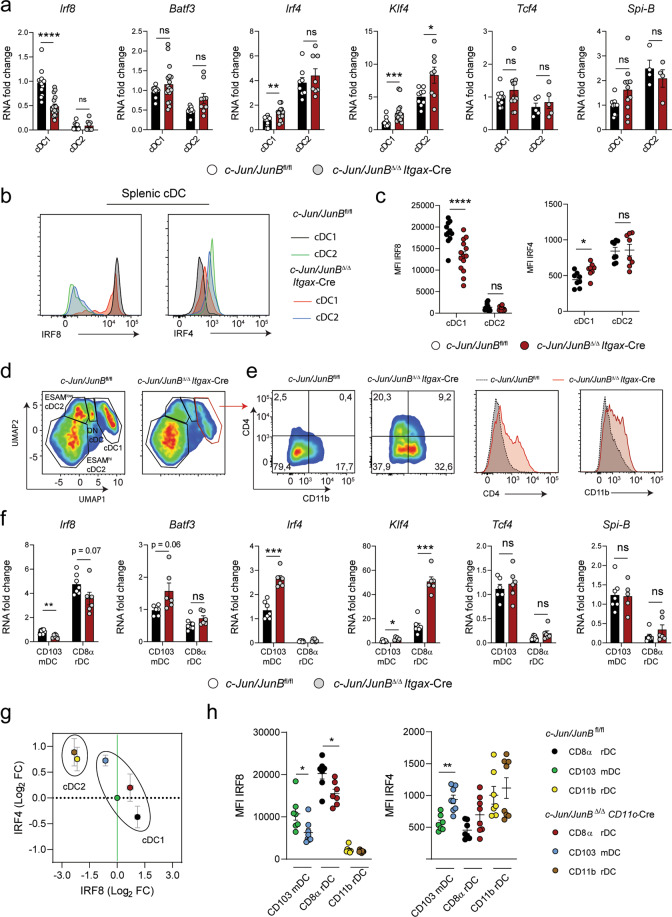
c-Jun and JunB maintain cDC1 identity. **a** Splenic CD8α cDC1 (CD3^-^CD19^-^BST-2^-^Siglec-H^-^CD11c^+^I-A/I-E^+^XCR1^+^CD8α^+^) from *c-Jun/JunB*^Δ/Δ^*Itgax-*Cre and *c-Jun/JunB*^fl/fl^ mice were sorted and RT-qPCR analysis of *Irf8, Batf3, Irf4, Klf4, Tcf4* and *Spi-B* mRNA was performed (*n* = 11–17 for cDC1 and 4–9 for cDC2 subgroups). **b** Splenic cDC (CD11c^+^I-A/I-E^+^) subsets cDC1 (XCR1^+^ cDC) and cDC2 (CD172a^+^ cDC) were analyzed for the protein expression of the transcription factors IRF4 and IRF8 by intracellular flow cytometry. Shown are representative histograms of IRF4 and IRF8 expression in cDC1 and cDC2 from indicated mice. **c** Graph depicts the mean fluorescence intensity (MFI) of IRF4 and IRF8 in splenic cDC subsets defined in (**b**). **d** UMAP analysis of splenic cDCs (CD11c^+^I-A/I-E^+^) acquired by flow cytometry, including the surface markers CD4, CD8α, CD11b, CD172a, ESAM, and XCR1 in *c-Jun/JunB*^Δ/Δ^*Itgax*-Cre and *c-Jun/JunB*^fl/fl^ mice. Samples were concatenated (*n* = 4 per genotype) to perform the analysis. **e** Expression of CD4 and CD11b on cells within the cDC1 population, as defined by UMAP in (**d**), was analyzed. Flow cytometry plots (left) and histograms (right) are shown. **f** Lymph-node resident (CD3^-^CD19^-^NK1.1^-^TCRβ^-^CD11c^+^I-A/I-E^int^XCR1^+^CD8α^+^) and migratory cDC1 (CD3^-^CD19^-^NK1.1^-^TCRβ^-^CD11c^+^I-A/I-E^high^XCR1^+^CD103^+^) from *c-Jun/JunB*^Δ/Δ^*Itgax-*Cre and *c-Jun/JunB*^fl/fl^ mice were sorted and RT-qPCR analysis of *Irf8, Batf3, Irf4, Klf4, Tcf4* and *Spi-B* mRNA was performed (*n* = 6–7). **g** Lymph-node CD8α rDC (CD11c^+^I-A/I-E^int^ XCR1^+^CD11b^-^), CD103 mDC (CD11c^+^I-A/I-E^high^XCR1^+^CD11b^-^CD103^+^) and CD11b rDC (CD11c^+^I-A/I-E^int^ XCR1^-^CD11b^+^) were analyzed by intracellular flow cytometry for IRF4 and IRF8 protein expression in indicated mice. Shown is the log_2_ Fold change of the MFI for IRF4 and IRF8 relative to the *c-Jun/JunB*^fl/fl^ CD103 mDC subset, which is set to 0. (*n* = 7 *c-Jun/JunB*^fl/fl^ and *n* = 8 *c- Jun/JunB*^Δ/Δ^*Itgax-*Cre). **h** Graph depicts the MFI of IRF4 and IRF8 in skin-draining lymph node cDC subsets as defined in (**g**). Data are representative of 2–4 independent experiments. Dots indicate number of individual mice per experimental group. Error bars represent mean ± SEM. Statistical significance was determined by multiple *t*-test with the Holm–Šídák method (**a**, **c**, **f**, **h**). ****P < 0.0001, ***P < 0.001, **P < 0.01, * *P* < 0.05, ns > 0.05.

Next, we investigated whether the functional impairment of the remaining CD8α splenic cDC1 was caused by an altered cDC1 lineage specification. Interestingly, the expression of TFs essential for cDC2 development and function, namely *Irf4* and *Klf4*, were significantly increased in c-Jun/JunB-deficient cDC1, although expression levels were lower compared to cDC2 (Fig. 7a). Despite comparable levels of *Batf3* and TFs involved in pDC development (*Tcf4* and *Spi-B), Irf8* was significantly reduced in cDC1 lacking c-Jun/JunB (Fig. 7a). Similarly, c-Jun/JunB-deficient cDC1^FL^ showed upregulated *Irf4* and *Klf4* transcript levels, which remained high during the course of differentiation compared to control cells, while pDC- and cDC1-defining TFs were unchanged, except for *Irf8* in pre-cDCs (Supplementary Fig. 6a-c). Consistently, splenic cDC1 lacking c-Jun/JunB showed significantly elevated IRF4 and reduced IRF8 protein levels (Fig. 7b-c).

Besides TFs, cellular identity is defined by exclusive expression of specific cell surface markers. Our RNA-Seq data showed an elevated expression of surface marker transcripts on c-Jun/JunB deficient cDC1^FL^ that are normally found on cDC2 or pDCs (Supplementary Fig. 7a). UMAP analysis, revealed a reduction of cDC1 and a shift to a cDC2 phenotype in *c-Jun/JunB*^Δ/Δ^ *Itgax*-Cre mice (Fig. 7d). Analysis of the cDC1 population, defined in the UMAP space, showed an elevated expression of cDC2 specific markers (CD4 and CD11b) on c-Jun/JunB deficient cDC1 (Fig. 7e). Correct population clustering by UMAP and increased surface expression of CD4 and CD11b was confirmed by conventional gating (Supplementary Fig. 7b-d). Next, we sorted CD8α rDC and CD103 mDC from sd-LN to better characterize the TF profile in these two population. Similar to splenic CD8α cDC1 resident as well as migratory cDC1 showed a shift to a cDC2 phenotype with reduced *Irf8*, but increased *Irf4* and *Klf4* mRNA expression, whereas the pDC TFs *Tcf4* and *Spi-B* were unchanged (Fig. 7f). Analysis of IRF4 and IRF8 protein by flow cytometry confirmed this altered cDC2-like phenotype (Fig. 7g, h). Lastly, deletion of *c-Jun* and *JunB* in sorted cDC1 and cDC2 was confirmed by quantitative RT-PCR (Supplementary Fig. 7e-f).

In conclusion, in this study we discovered a novel TF pair, c-Jun/JunB, required for CD8α cDC1 development, function, and maintenance of their identity.

## Discussion

cDC1 differentiation and identity is controlled by a complex network of linage-determining TFs. So far IRF8, ID2, NFIL3 and Batf3 have been reported as critical regulators at different stages of CD8α and CD103 cDC1 development [7]. In this study, we identify the AP-1 family member’s c-Jun and JunB as two novel TFs essential for the development and function of the CD8α cDC1 lineage.

cDC1 develop from progenitor cells residing in the BM (CDPs), and differentiate in the tissue from a transitory, immediate progenitor population, the pre-cDC1 [7]. Our results demonstrate that the combined deletion of c-Jun and JunB in  CD11c^+^ cells induces a severe defect in the specification of pre-cDCs to CD8α cDC1. Importantly, other immune cell subsets developed normally in this mouse model, emphasizing c-Jun/JunB as specific-lineage determining TFs for CD8α cDC1 commitment.

In single c-Jun or JunB knock-out mice no effect on the CD8α cDC1 pool was observed. Thus, c-Jun and JunB seem to either compensate for each other or cooperate by a currently unclear mechanism. Our data demonstrate for the first time such a non-redundant function for c-Jun and JunB in immune cells, but similar observations have been made in keratinocytes and hair follicle stem cells [25, 53].

Batf3 was the first AP-1 TF shown to be essential for CD8α cDC1 [13] and, depending on the mouse background, for CD103 cDC1 development [28, 42]. 129 SvEv-*Batf3*^*−/−*^ mice lack CD8α and CD103 cDC1s under homeostatic conditions [13, 42], whereas in a C57BL/6-background only a reduced splenic CD8α cDC1 population was observed [54, 55]. In our study both GEMM models, *c-Jun*/*JunB*^Δ/Δ^*Mx*-Cre and *Itgax*-Cre, are in a mixed (129Sv × C57BL/6) background and show only a reduction in lymphoid-resident CD8α cDC1. It thus remains to be investigated, whether the unaltered CD103 cDC1 pool is due to the mixed background.

Pathogenic infections or transplantations can restore the cDC1 pool independently of Batf3 [28, 56]. Batf3 can be replaced by its family members Batf or Batf2 during infections with intracellular pathogens [28]. Batf has been described to hetero-dimerize with c-Jun, JunB or JunD in vitro, whereas for Batf2 and Batf3 only hetero-dimerization with c-Jun has been tested [26, 57, 58]. Our findings suggest that both c-Jun and JunB are non-redundant partners for Batf3 in the lineage decision of pre-cDCs. Additional studies are required to test, if c-Jun and JunB are also essential interaction partners for Batf or Batf2 to mediate compensatory DC development. In *Batf3*^−/−^ mice, also T-cell function is affected [59]. Therefore, *c-Jun/JunB*^Δ/Δ^*Itgax-*Cre mice could be used as an alternative mouse model to study CD8α cDC1 biology under homeostatic and inflammatory conditions.

The classic DNA-binding element bound by AP-1 proteins is the TPA-responsive element (TRE) [16], but cooperation with IRF proteins expands the repertoire to AICE elements [28]. Although a proportion of the DEGs in c-Jun/JunB deficient cDC1 was overlapping with Batf3, the majority of genes were exclusive and associated with cell function, signaling and metabolism. Future studies could elucidate the role of classical elements (TRE) bound by AP-1 dimers compared to AICE elements recognized by AP-1-IRF hetero-trimers for cDC1 biology.

Importantly, our findings emphasize that not only deletion of c-Jun/JunB in hematopoietic cells, but also in other cell types affects cDC1 development. In a recent study, G-CSF, which is a direct transcriptional target of JunB in keratinocytes [44], has been shown to interrupt IRF8 dependent cDC1 development in the BM [45]. Here, we show that neutralization of G-CSF in *c-Jun/JunB*^Δ/Δ^*Mx*-Cre mice partially rescued cDC1 numbers in the spleen. Future work should examine this novel extrinsic mechanism of cDC1 differentiation in more detail.

c-Jun/JunB deficient cDC1 have reduced *Irf8* expression, despite normal levels of *Batf3*, and show a shift towards a cDC2 phenotype. Similarly, *Batf3*^*−/−*^-deficient pre-CD8 DC cannot maintain IRF8 expression and divert to cDC2 [14].

*Irf8* expression was also reduced in c-Jun/JunB deficient CD103 mDC, but their frequency was normal. Given the dependence of this DC subset on GM-CSF [47], further studies are needed to clarify its role in promoting CD103 mDC development/survival in the absence of c-Jun/JunB.

Collectively, our findings identify a key role for c-Jun/JunB in cDC1 development, identity and function through extrinsic and intrinsic mechanisms.

## Supplementary information

Supplementary Figures

Supplementary Table 1

Supplementary Table 2

Supplementary Table 3

Supplementary Table 4

Supplementary Table 5

## Data Availability

The RNA‐Seq dataset has been deposited to GEO under the accession number GSE156484.
