# Supplementary material for: The AP-1 transcription factors c-Jun and JunB are essential for CD8α conventional dendritic cell identity
Source: Cell Death Differ. 2021 Mar 23;28(8):2404–20. doi: 10.1038/s41418-021-00765-4 (PMC8329169; doi:10.1038/s41418-021-00765-4)
Supplement: Supplementary file 1 — Supplementary Figures [file 41418_2021_765_MOESM1_ESM.pdf]

Supplementary Figures of the research article entitled:

The AP-1 transcription factors c-Jun and JunB are essential for CD8α conventional dendritic cell identity *by Novoszel, Drobits et al*.

Table of contents:

1. **Supplementary Figure Legends**

2. **Supplementary Figures**

1. Supplementary Figure Legends

Supplementary Figure 1. c-Jun and JunB show prominent expression in cDCs, but are individually dispensable for cDC development

a Heat map shows expression of indicated TFs across the listed immune cells. MyGeneSet tool (http://rstats.immgen.org/MyGeneSet_New/index.html) was used to generate a heat map with microarray V1 data as an input.

b Expression of c-Jun and JunB was analyzed in indicated immune cell populations *in vivo*. Data were obtained from a published gene expression microarray [GEO: microarray data GSE66899]. Fold change is shown relative to the CDP population.

c Splenic cDCs ($CD11c^{+}$I-A/$I\text{-}E^{+}$) from *c-Jun*$^{fl/fl}$ and *c-Jun*$^{\Delta/\Delta}$ *Mx*-Cre mice were analyzed for CD8α cDC1 ($CD8\alpha^{+}$ $CD11b^{-}$cDC) and CD11b cDC2 ($CD8\alpha^{-}$ $CD11b^{+}$ cDC) frequency and absolute numbers (d)**.**

e Representative flow cytometry plots, frequencies and absolute numbers (f) of splenic cDCs, CD8α cDC1 and CD11b cDC2 as defined in (c) of *JunB*$^{fl/fl}$ and *JunB*$^{\Delta/\Delta}$ *Mx*-Cre mice.

Data are representative of 2-4 independent experiments. Flow cytometric plots are pre-gated on single, live cells. Dots indicate number of individual mice per experimental group. Error bars represent mean ± SEM. Statistical significance was determined by One-Way ANOVA with Tukey posttest (b) and unpaired two-tailed Student´s *t*-test (d, f). ns > 0.05, * $P < 0.05$, ** $P < 0.01$.

Supplementary Figure 2. Immune-cell profile of *c-Jun/JunB* $^{\Delta/\Delta}$*Mx*-Cre mice

a Representative flow cytometry plots show the gating strategy to identify splenic myeloid cells ($CD11b^{+}$Ly6-C/G^{+}), splenic T cells (TCR-β^{+}) and splenic B cells ($CD19^{+}$) in *c-Jun/JunB*$^{\Delta/\Delta}$Mx-Cre and control mice (*c-Jun/JunB* $^{fl/fl}$).

b Frequency and total number of splenic myeloid cells, T cells and B cells identified as described in (a) is shown.

c Representative images of hematoxylin and eosin stained back skin. Skin inflammation induced after short-term deletion of c-Jun/JunB in the *Mx*-Cre mouse model is indicated by arrows showing epidermal thickening (acanthosis) and abscess formation. Magnification: 20x, Bright-Field Image.

d BM from *c-Jun/JunB*$^{fl/fl}$ and *c-Jun/JunB*$^{\Delta/\Delta}$*Mx*-Cre mice was analyzed by flow cytometry for GMPs (granulocyte macrophage progenitor, Lin^-Sca1^- $CD11b^-CD135^-CD16/32^+CD117^{High}$), CMPs (common myeloid progenitor, Lin^-Sca1^- $CD11b^-CD16/32^+CD135^+CD117^{High}$), CDPs (common dendritic progenitors, Lin^-Sca1^- $CD11b^-$ $CD16/32^-CD135^+$ $CD117^{int}$), and pre-cDCs (Lin^-Sca1^- $CD11b^-$ $CD11c^+CD135^+$ $CD117^{low}$).

e BM progenitors defined in (d) are shown as percentage of live, single cells.

f BM was analyzed by flow cytometry for B cells ($CD19^+$), NK cells ($NK1.1^+$) and T cells ($TCR\beta^+$) expressing CD4 or CD8.

g RT-qPCR analysis of *c-Jun* and *JunB* mRNA expression was performed on sorted progenitors, pre-cDC^{FL} and cDC^{FL} to confirm deletion in FLT3L supplemented *c-Jun/JunB*$^{\Delta/\Delta}$*Mx*-Cre BM cultures.

h Representative flow cytometry plots show pDCs^{FL} ($B220^+CD11c^+$) and the cDC^{FL} ($B220^-CD11c^+$) subsets cDC1^{FL} ($CD24^+$ cDC^{FL}) and cDC2^{FL} ($CD172a^+$ cDC^{FL}) in FLT3L supplemented BM cultures of *c-Jun/JunB* $^{fl/fl}$ and *c-Jun/JunB*$^{\Delta/\Delta}$Mx-Cre mice at day 8.

i Representative histograms show expression of CD24 and CD172a on cDC^{FL} cells from (h).

j Non-adherent cells from (h) were stimulated with poly I: C (16h) and expression of CD86 was analyzed on cDC^{FL}. Representative histograms are shown.

Data are representative of 1 -2 experiments. Dots indicate number of individual mice per experimental group. Error bars represent mean ± SEM. Statistical significance was determined by unpaired Student´s *t*-test (b, e, and g). ns > 0.05, * $P < 0.05$, ** $P < 0.01$, *** $P < 0.001$.

Supplementary Figure 3. Extrinsic factors, regulated by c-Jun/JunB, influence CD8α cDC1 development

a Spleen from *c-Jun/JunB*$^{fl/f}$, *c-Jun/JunB*$^{\Delta/\Delta}$*K5*-Cre^{ER}, *c-Jun/JunB*$^{\Delta/\Delta}$*Mx*-Cre was analyzed for the cDC (CD11c^{+}I-A/I-E^{+}) subsets CD8α cDC1 and CD11b cDC2. Deletion of c-Jun and JunB in the *c-Jun/JunB*$^{\Delta/\Delta}$*K5*-Cre^{ER} mouse model was induced by intraperitoneal injection of Tamoxifen (1mg, 5 consecutive days) and mice were analyzed 14 days after Tamoxifen treatment started, when a psoriasis-like skin phenotype was present.

b Frequency of *c-Jun/JunB* $^{fl/f}$, *c-Jun/JunB*$^{\Delta/\Delta}$*K5*-Cre^{ER}, *c-Jun/JunB*$^{\Delta/\Delta}$*Mx*-Cre splenic CD8α cDC1 as defined in (a) is shown.

c Total number of splenic CD8α cDC1 in the epidermal (*K5*-Cre^{ER}) and hematopoietic (*Mx*-Cre) mouse model as defined in (a) is shown.

d Representative flow cytometry plots show the cDC (CD11c^{+}I-A/I-E^{+}) subsets CD8α cDC1 and CD11b cDC2 in spleen of wild-type mice treated topically with Imiquimod (IMQ) for 7 days.

e Frequency of CD8α cDC1 as defined in (d) is shown.

Data are representative of 2 independent experiments. Dots indicate number of individual mice per experimental group. Error bars represent mean ± SEM. Statistical significance was determined by unpaired Student´s *t*-test. * $P < 0.05$.

Supplementary Figure 4. cDC progenitor populations develop normally in *c- Jun/JunB*$^{\Delta/\Delta}$*Itgax*-Cre mice

a Immune cells in spleen of *c-Jun/JunB*$^{fl/fl}$ and *c-Jun/JunB*$^{\Delta/\Delta}$*Itgax*-Cre were analyzed by flow cytometry. B cells are CD19^{+}, NK cells NK1.1^{+}, pDCs BST-2^{+} and B220^{+} and T cells are TCR-β^{+} and CD4^{+} or CD8^{+}.

b Graph shows frequency of CD24^{+}, DEC-205^{+} or XCR1^{+} splenic cDCs (CD11c^{+}I-A/I-E^{+}) in *c-Jun/JunB*$^{fl/fl}$ and *c-Jun/JunB*$^{\Delta/\Delta}$*Itgax*-Cre mice.

c BM of *c-Jun/JunB*$^{fl/fl}$ and *c-Jun/JunB*$^{\Delta/\Delta}$*Itgax*-Cre mice was analyzed by flow cytometry for GMP, CMP, CDP and pre-cDC cells, gated as in Supplementary Fig. 2d.

d Representative flow cytometry plots of pre-cDCs ($Lin^-CD11c^+CD135^+CD172a^-MHCII^-$), grouped into pre-cDC 1 (Siglec-H^-Ly6C^-) and pre-cDC 2 (Siglec-H^-Ly6C^+), in spleen and BM of the indicated genotypes is shown.

e Frequencies of BM and splenic pre-cDC 1 and pre-cDC 2 analyzed according to (d) are shown.

f Representative flow cytometry plots show CDP^{FL} ($CD117^{low\text{-}int}CD115^+CD11c^-MCHII^-$) and pre-$cDC^{FL}$ ($CD117^{low\text{-}int}CD115^+CD11c^+MCHII^-$) populations in FLT3L supplemented BM cultures from *c-Jun/ JunB* $^{fl/fl}$ and *c-Jun/JunB*$^{\Delta/\Delta}$*Itgax*-Cre mice on day 7.

g Frequency and absolute number of CDP^{FL}, pre-cDC^{FL} and pDC^{FL} populations was analyzed by flow cytometry on the indicated days in FLT3L supplemented BM cultures from *c-Jun/JunB*$^{fl/fl}$ and *c-Jun/JunB*$^{\Delta/\Delta}$*Itgax*-Cre mice. pDC^{FL} were defined as $B220^+CD11c^+$ and CDP^{FL}, pre-cDC^{FL} were gated as in (f). n = 6-12 for both genotypes at every time-point.

Data are representative of 2- 4 independent experiments. Dots indicate number of individual mice per experimental group. Error bars represent mean ± SEM. Statistical significance was determined by unpaired Student´s *t*-test. ns > 0.05, * $P < 0.05$, ** $P < 0.01$, *** $P < 0.001$.

Supplementary Figure 5. c-Jun and JunB control cDC1^{FL} function, but are dispensable for CD103 cDC development in non-lymphoid tissues

a-b The CD103 cDC1 population in the lung (a) and colon (b) was analyzed by flow cytometry. Plots on the left show $XCR1^+$ $CD103^+$ cDC1 cells. Plots were pre-gated on $CD45^+CD11c^+$I-A/I-E^+ cells. Graph on the right gives frequency and cell number of $CD103^+$ cDC1 in the indicated tissue.

c FLT3L-derived, non-adherent cells were recovered on day 8 of culture and stimulated with the TLR3 ligand poly I: C (1 µg/mL) for 5h in the presence of Brefeldin A. Intracellular flow cytometry was performed to analyze IL-12p40 production by $cDC1^{FL}$ ($CD24^+$ cDC^{FL}). Representative plots shown on the left are pre-gated on single, live, and cDC^{FL} ($B220^-CD11c^+$). Graph on the right shows percentage of IL-12p40 producing $cDC1^{FL}$ to live, single cells.

d Surface expression of CD80 and CD86 was analyzed on cDC1^{FL} by flow cytometry after stimulation of non-adherent cells with TLR3 ligand poly I: C (1 µg/mL) for 16h on day 8 of FLT3L-supplemented *c-Jun/JunB*$^{fl/fl}$ and *c-Jun/JunB*$^{\Delta/\Delta}$*Itgax*-Cre BM culture. Representative histograms (left) and mean fluorescence intensity (MFI) (right) of CD80 and CD86 expression on cDC1^{FL} are shown.

Data are representative of 1- 4 independent experiments. Dots indicate number of individual mice per experimental group. Error bars represent mean ± SEM. Statistical significance was determined by unpaired Student´s *t*-test (a, b) or one-way ANOVA with Tukey posttest (c, d). ns > 0.05, ** $P < 0.01$, *** $P < 0.001$.

Supplementary Figure 6. Transcriptional signature of c-Jun/JunB deficient cDC1^{FL}

a Heat map of differentially regulated transcription factor transcripts in control versus c-Jun/JunB-deficient cDC1^{FL} from RNA-Seq data generated as described in Methods section. Gene Ontology annotation GO: 0003677 was applied to filter for TFs in differentially expressed genes (DEG) ($P < 0.05$, $\log_2$FC ≥ 1). Color indicates up-regulated DEGs with highest expression in cDC1 (red), cDC2 (blue) or pDC (green) as analyzed by MyGeneSet tool (http://rstats.immgen.org/MyGeneSet_New/index.html) with the ImmGen ULI RNA-Seq dendritic cell population as input.

b Graphs show RNA expression of selected TFs essential for cDC1, cDC2 and pDC development in *c-Jun/JunB*$^{fl/fl}$ and *c-Jun/JunB*$^{\Delta/\Delta}$*Itgax*-Cre cDC1^{FL} RNA-Seq samples. Dots indicate numbers of individual mice per experimental group.

c RT-qPCR analysis of *Irf8*, *Batf3*, *Irf4*, *Klf4*, *c-Jun*, *JunB*, *Tcf4,* and *Spi-B* mRNA expression in FACS sorted progenitors, pre-cDCs^{FL}, cDC1^{FL} from FLT3L supplemented *c-Jun/JunB*$^{\Delta/\Delta}$*Itgax*-Cre and *c-Jun/JunB*$^{fl/fl}$ BM cultures (Day 8).

Data are representative of 2-4 independent experiments. Dots indicate number of individual mice per experimental group. Error bars represent mean ± SEM. Statistical significance was determined by

Benjamini-Hochberg method (a, b) or unpaired multiple *t*-test with the Holm-Šídák method (c). * *P* < 0.05, ** *P* < 0.01, *** *P* < 0.001.

Supplementary Figure 7. Cell-surface phenotype of c-Jun/JunB deficient cDC1

a Heat map shows differential expression of cell surface and membrane associated transcripts (GO: 0009986) in control versus c-Jun/JunB-deficient $cDC1^{FL}$ ($P < 0.05$, $log_2FC \geq 1$). Color indicates up-regulated DEGs with highest expression in cDC1 (red), cDC2 (blue) or pDCs (green) as analyzed by MyGeneSet tool (http://rstats.immgen.org/MyGeneSet_New/index.html) with the ImmGen ULI RNA-Seq dendritic cell population as input.

b Overlap of splenic cDC subsets defined by conventional flow cytometry and UMAP clustering. Samples (*n*= 4) were concatenated to perform the analysis.

c Surface expression of cDC2 specific markers (CD4, CD11b) was analyzed by flow cytometry on $cDC1^{FL}$ and is shown as mean fluorescence intensity (MFI). $cDC1^{FL}$ were generated from BM of *c-Jun/JunB*$^{fl/fl}$ and *c- Jun/JunB*$^{\Delta/\Delta}$*Itgax*-Cre mice supplemented with FLT3L for 9 days.

d Splenic cDC1 ($CD11c^+$ $I\text{-}A^+/I\text{-}E^+$ $XCR1^+$) from *c-Jun/JunB*$^{fl/fl}$ and *c-Jun/JunB*$^{\Delta/\Delta}$*Itgax*-Cre mice were analyzed for expression of CD4 and CD11b. Shown is the mean fluorescence intensity (MFI).

e Quantitative RT-PCR of *c-Jun* and *JunB* performed on cDC1 and cDC2 sorted from spleen of *c-Jun/JunB*$^{fl/fl}$ and *c-Jun/JunB*$^{\Delta/\Delta}$*Itgax*-Cre mice as described in Fig 7a.

f Quantitative RT-PCR of *c-Jun* and *JunB* performed on resident and migratory cDC1 sorted from lymph-nodes of *c-Jun/JunB*$^{fl/fl}$ and *c-Jun/JunB*$^{\Delta/\Delta}$*Itgax*-Cre mice as described in Fig. 7f.

Data are representative of 2-6 independent experiments. Dots indicate number of individual mice per experimental group. Error bars represent mean ± SEM. Statistical significance was determined by Benjamini-Hochberg method (a) or unpaired Student´s *t*-test (c, d) or unpaired multiple *t*-test with the Holm-Šídák method (e, f) . * *P* < 0.05, ** *P* < 0.01, *** *P* < 0.001.

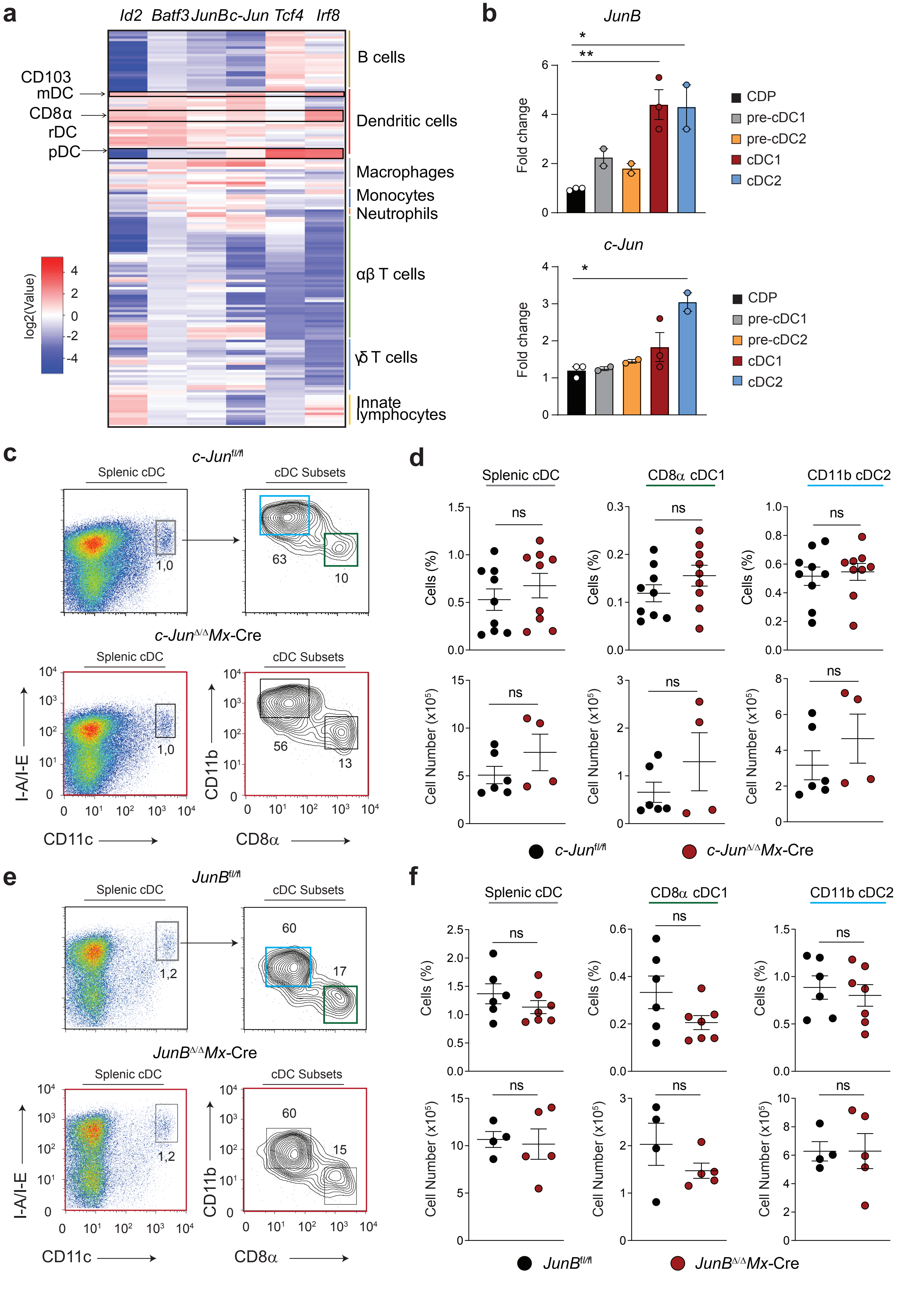

Supplementary Fig. 1

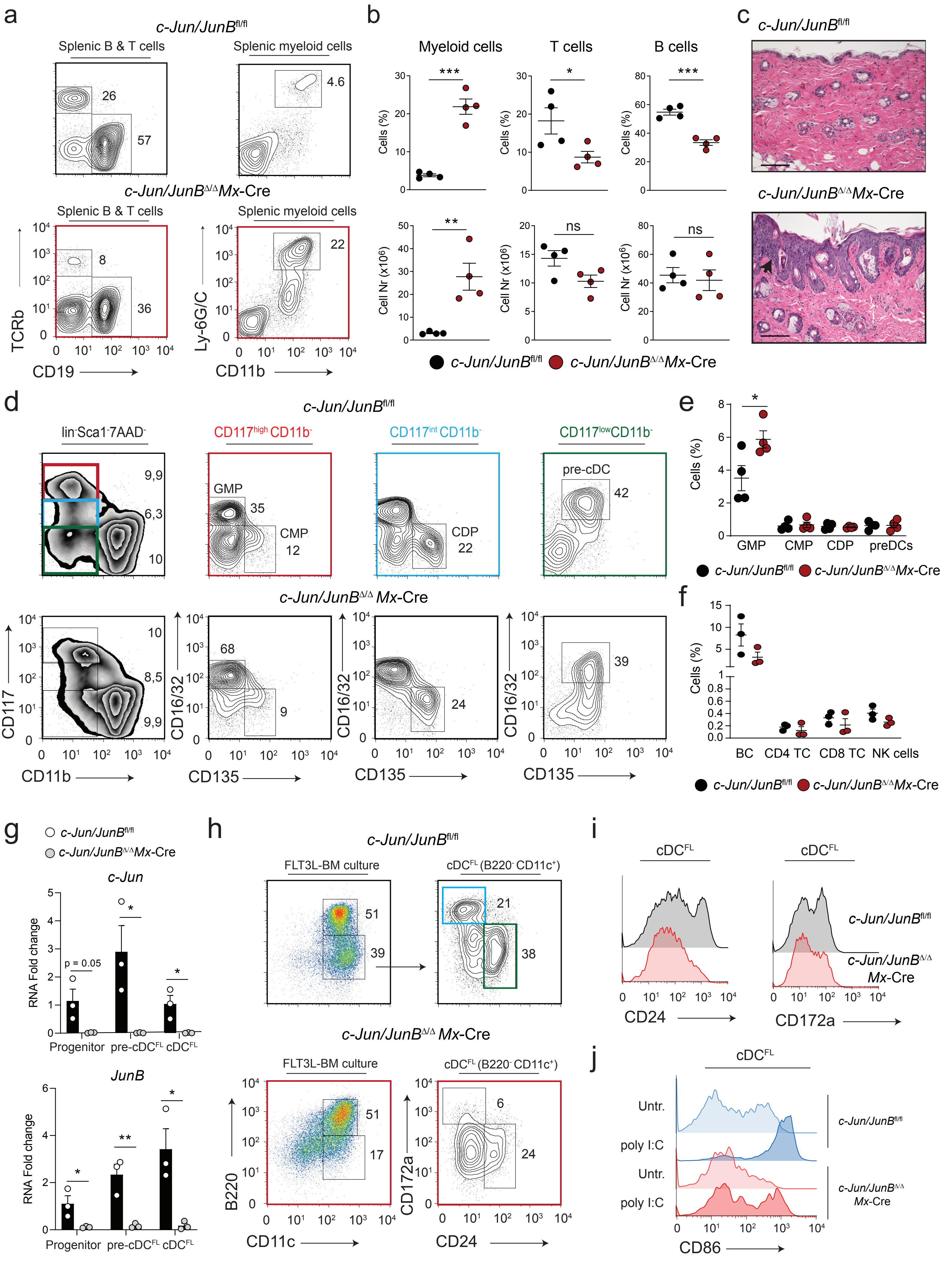

Supplementary Fig. 2

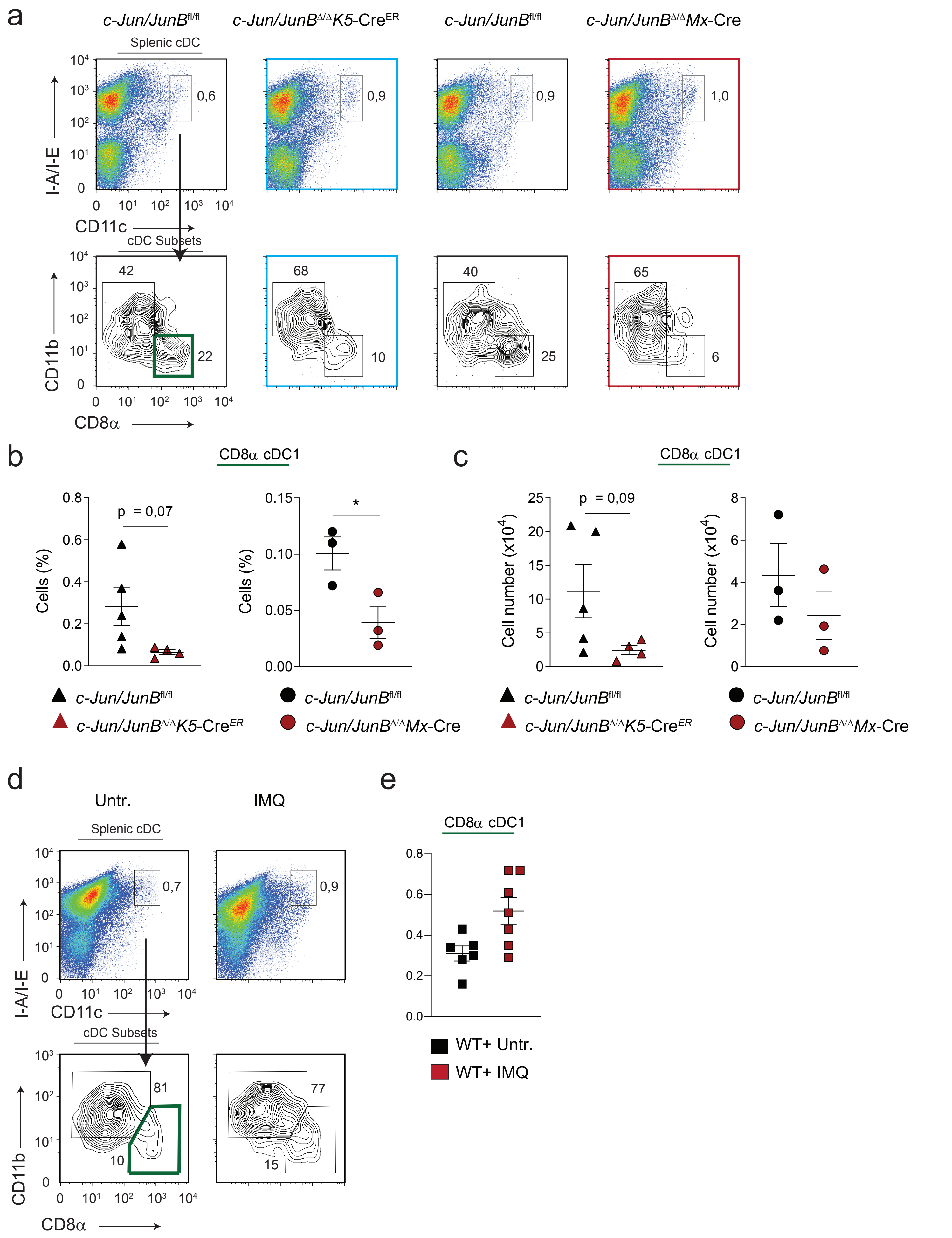

Supplementary Fig. 3

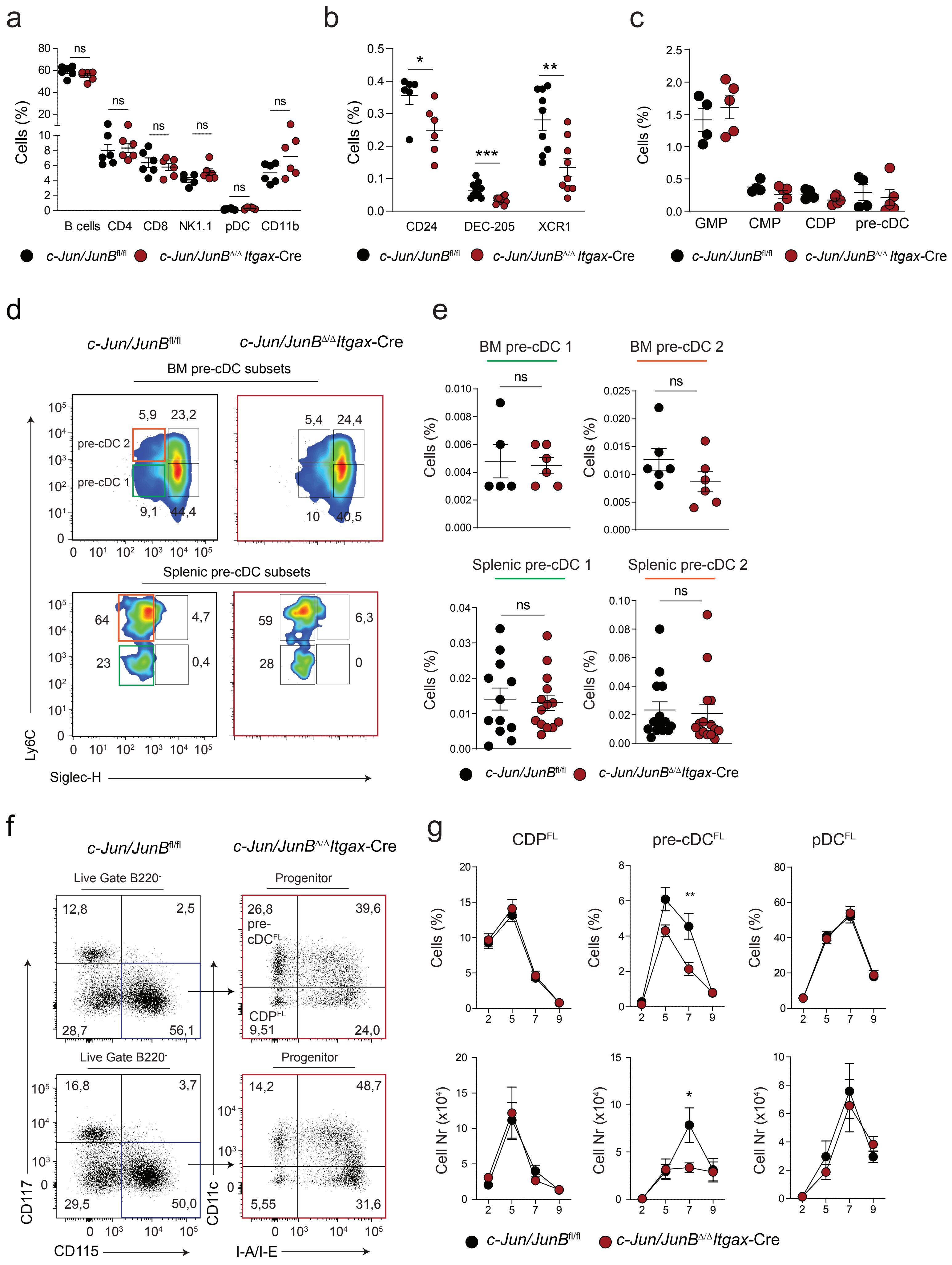

Supplementary Fig. 4

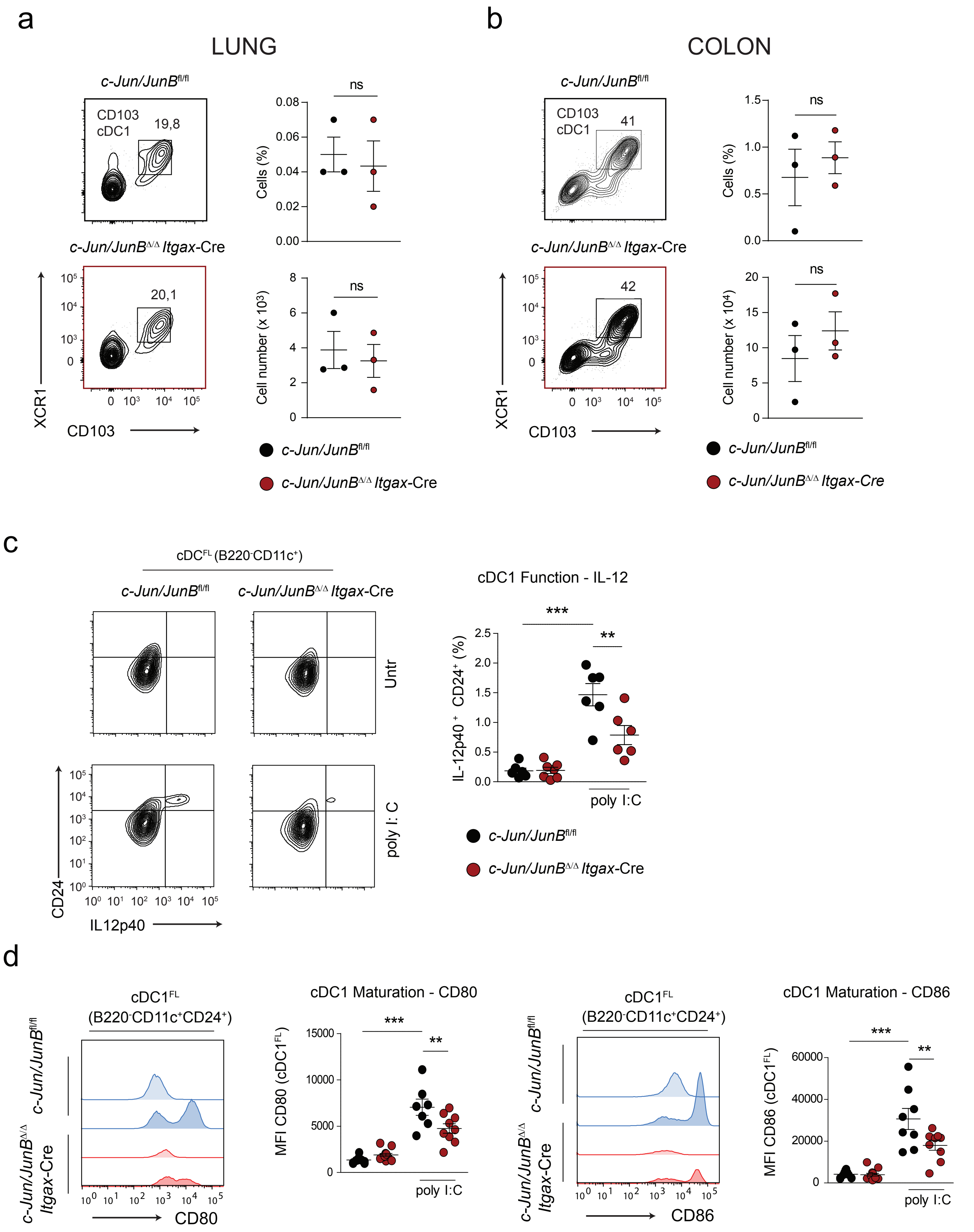

Supplementary Fig. 5

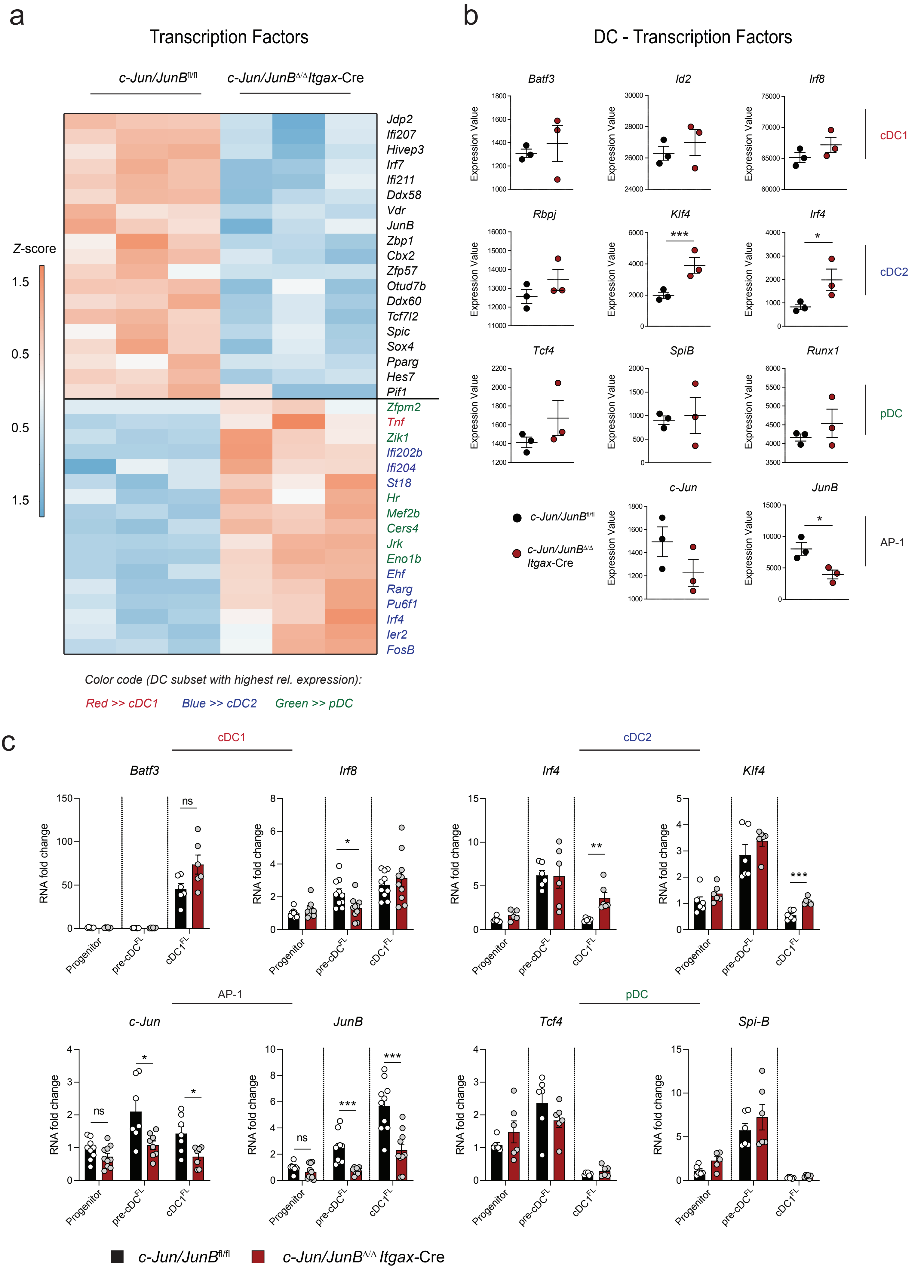

Supplementary Fig. 6

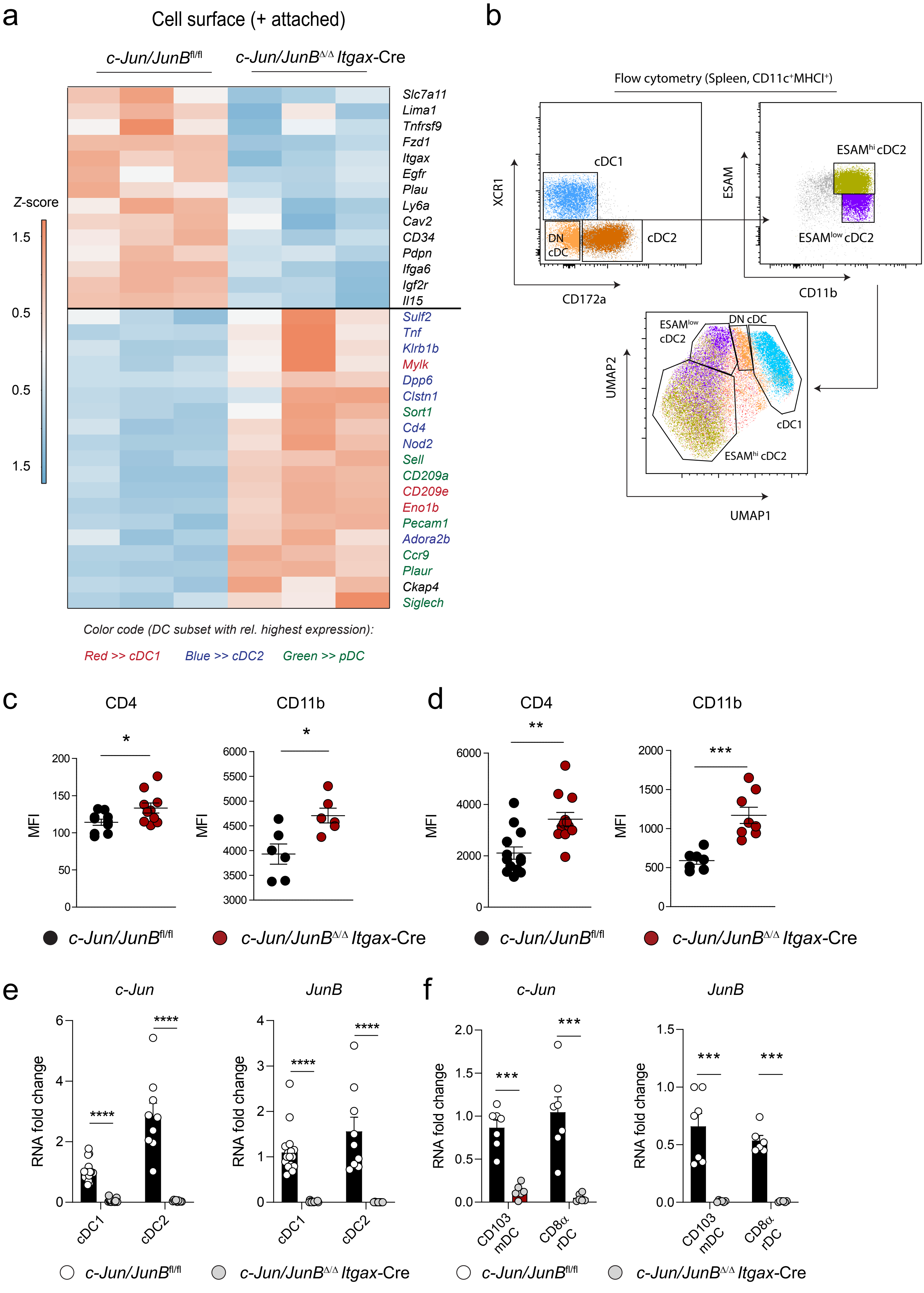

Supplementary Fig. 7
