# Supplementary material for: The AP-1 transcription factors c-Jun and JunB are essential for CD8α conventional dendritic cell identity
Source: Cell Death Differ. 2021 Mar 23;28(8):2404–20. doi: 10.1038/s41418-021-00765-4 (PMC8329169; doi:10.1038/s41418-021-00765-4)
Supplement: Supplementary file 2 — Supplementary Table 1 [file 41418_2021_765_MOESM2_ESM.docx]

## **Supplementary Table 1.** Antibodies for flow cytometry

## The following table lists the antibodies that were used in flow cytometry experiments.

| **Antibody** | **Clone** | **Company** | **Dilution** |
| --- | --- | --- | --- |
| B220 | RA3-6B2 | BioLegend | 0.25 µg/10^6^ cells |
| BST-2 | 927 | BioLegend | 0.25 µg/10^6^ cells |
| CD3ε | 145-2C11 | BioLegend | 0.25 µg/10^6^ cells |
| CD4 | GK 1.5 | BioLegend | 0.25 µg/10^6^ cells |
| CD8α | 53-6.7 | BioLegend | 0.25 µg/10^6^ cells |
| CD11b | M1/70 | BioLegend | 0.25 µg/10^6^ cells |
| CD11c | N418 | BioLegend | 0.25 µg/10^6^ cells |
| CD16/32 | 93 | BioLegend | 0.25 µg/10^6^ cells |
| CD19 | 6D5 | BioLegend | 0.25 µg/10^6^ cells |
| CD24 | M1/69 | BioLegend | 0.25 µg/10^6^ cells |
| CD45 | 30-F11 | BioLegend | 0.25 µg/10^6^ cells |
| CD64 | X54-5/7.1 | BioLegend | 0.25 µg/10^6^ cells |
| CD80 | 16-10A1 | BioLegend | 0.25 µg/10^6^ cells |
| CD86 | GL-1 | BioLegend | 0.25 µg/10^6^ cells |
| CD103 | M290 | BD Biosciences | 0.25 µg/10^6^ cells |
| CD115 | AFS98 | BioLegend | 0.25 µg/10^6^ cells |
| CD117 | 2B8 | BioLegend | 0.25 µg/10^6^ cells |
| CD135 | A2F10 | BioLegend | 0.25 µg/10^6^ cells |
| CD172a | P84 | BioLegend | 0.25 µg/10^6^ cells |
| CD205 (DEC-205) | NLDC-145 | BioLegend | 0.25 µg/10^6^ cells |
| ESAM | 1G8/ESAM | BioLegend | 0.25 µg/10^6^ cells |
| IL-12 p40 | C15.6 | BioLegend | 0.25 µg/10^6^ cells |
| IRF4 | 3E4 | eBioscience | 0.25 µg/10^6^ cells |
| IRF8 | V3GYWCH | eBioscience | 0.25 µg/10^6^ cells |
| Ly-6C | HK 1.4 | BioLegend | 0.25 µg/10^6^ cells |
| Ly-6G | 1A8 | BioLegend | 0.25 µg/10^6^ cells |
| Ly-6C/G | RB6-8C5 | BioLegend | 0.25 µg/10^6^ cells |
| MHC-II | M5/114.15.2 | BioLegend | 0.25 µg/10^6^ cells |
| NK 1.1 | PK136 | BioLegend | 0.25 µg/10^6^ cells |
| Sca-1 | D7 | BioLegend | 0.25 µg/10^6^ cells |
| Siglec-H | 551 | BioLegend | 0.25 µg/10^6^ cells |
| TCR β | H57-597 | BioLegend | 0.25 µg/10^6^ cells |
| XCR1 | ZET | BioLegend | 0.25 µg/10^6^ cells |
