# Supplementary material for: The AP-1 transcription factors c-Jun and JunB are essential for CD8α conventional dendritic cell identity
Source: Cell Death Differ. 2021 Mar 23;28(8):2404–20. doi: 10.1038/s41418-021-00765-4 (PMC8329169; doi:10.1038/s41418-021-00765-4)
Supplement: Supplementary file 3 — Supplementary Table 2 [file 41418_2021_765_MOESM3_ESM.docx]

## **Supplementary Table 2.** Murine RT-qPCR primers

## The following table lists primer pairs against indicated murine genes used in this study.

| **Murine Gene** | **Forward primer (5´-3´)** | **Reverse primer (5´-3´)** |
| --- | --- | --- |
| *Batf3* | GAGCCCCAAGGACGATGAC | GCTCTCGTGCTCCTCGTG |
| *c-Jun* | AAAACCTTGAAAGCGCAAAA | CGCAACCAGTCAAGTTCTCA |
| *Junb* | ATGTGCACGAAAATGGAACA | CCTGACCCGAAAAGTAGCTG |
| *Irf4* | CACAGCTCATGTGGAACCTCT | ACTCGTAGCCCCTCAGGAAA |
| *Irf8* | AACCGGCGGCAGGATG | ATTTTCCCAGATCAGCCCCG |
| *Klf4* | GACTAACCGTTGGCGTGAGG | CGGGTTGTTACTGCTGCAAG |
| *Spi-B* | TGCTCTGAACCACCATGCTT | CATGTAGAGTCAAGGCCCCC |
| *Tbp* | GGGGAGCTGTGATGTGAAGT | CCAGGAAATAATTCTGGCTCAT |
| *Tcf4* | GCAGGGATCTTGGGTCACAT | GAGACTCTGCTGGTGGCAA |
